# Supplementary figures and images for: CPT1A mediates radiation sensitivity in colorectal cancer
Source: eLife. 2024 Nov 28;13:RP97827. doi: 10.7554/eLife.97827 (PMC11604221; doi:10.7554/eLife.97827)

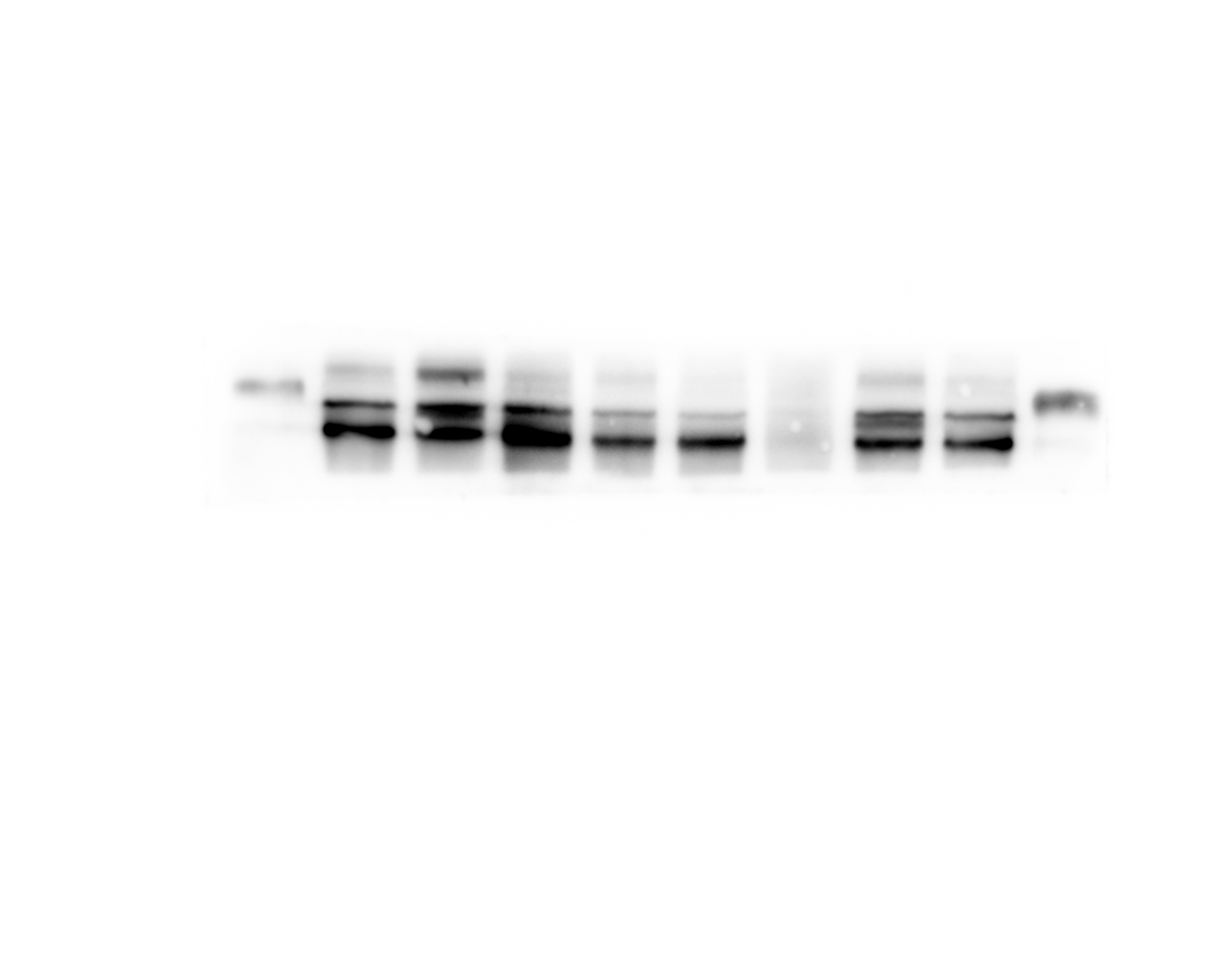

Supplement: Figure 1—source data 1. [file elife-97827-fig1-data1.zip › Figure 1-source data 1.1 .tiff]

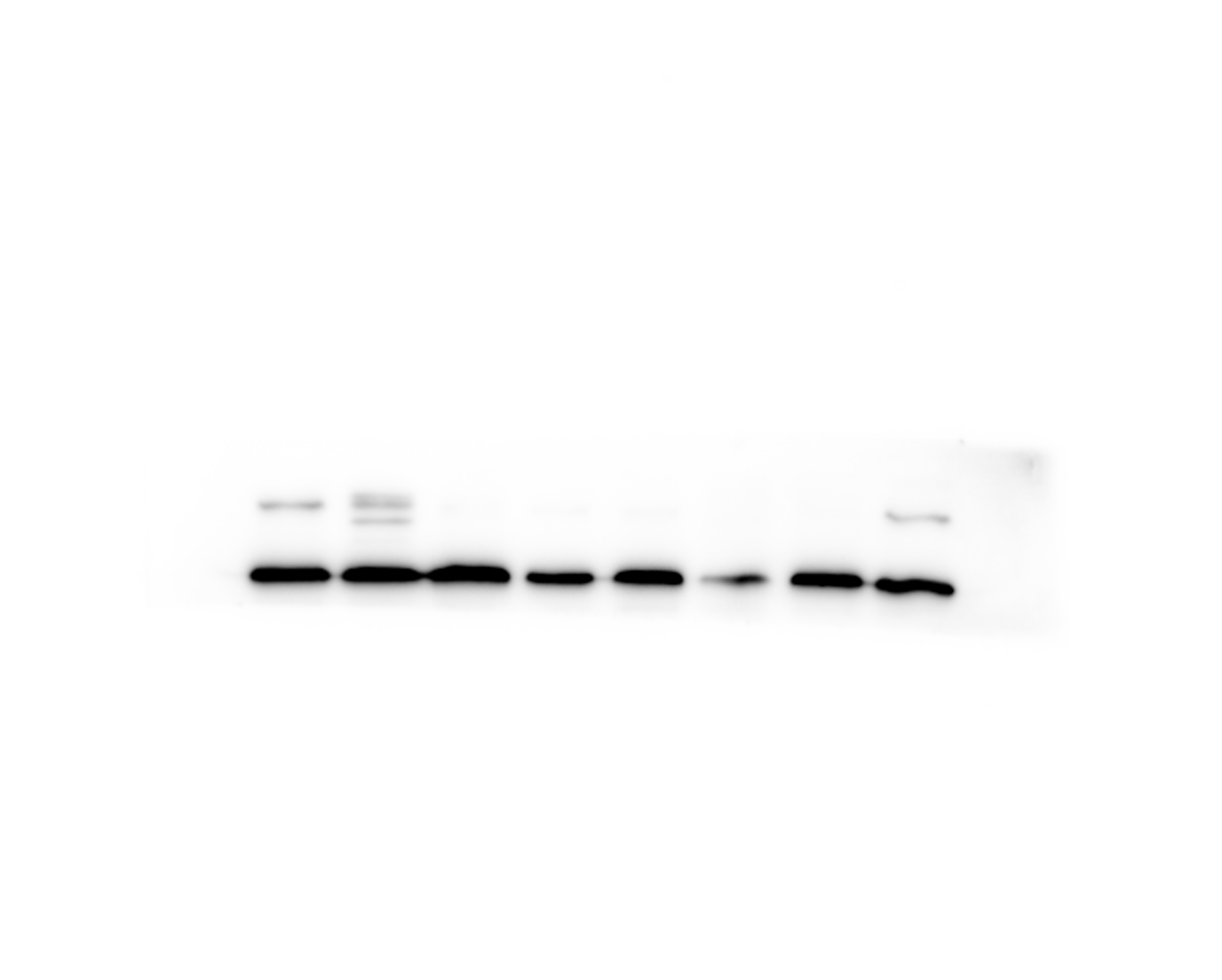

Supplement: Figure 1—source data 1. [file elife-97827-fig1-data1.zip › Figure 1-source data 1.2 .tiff]

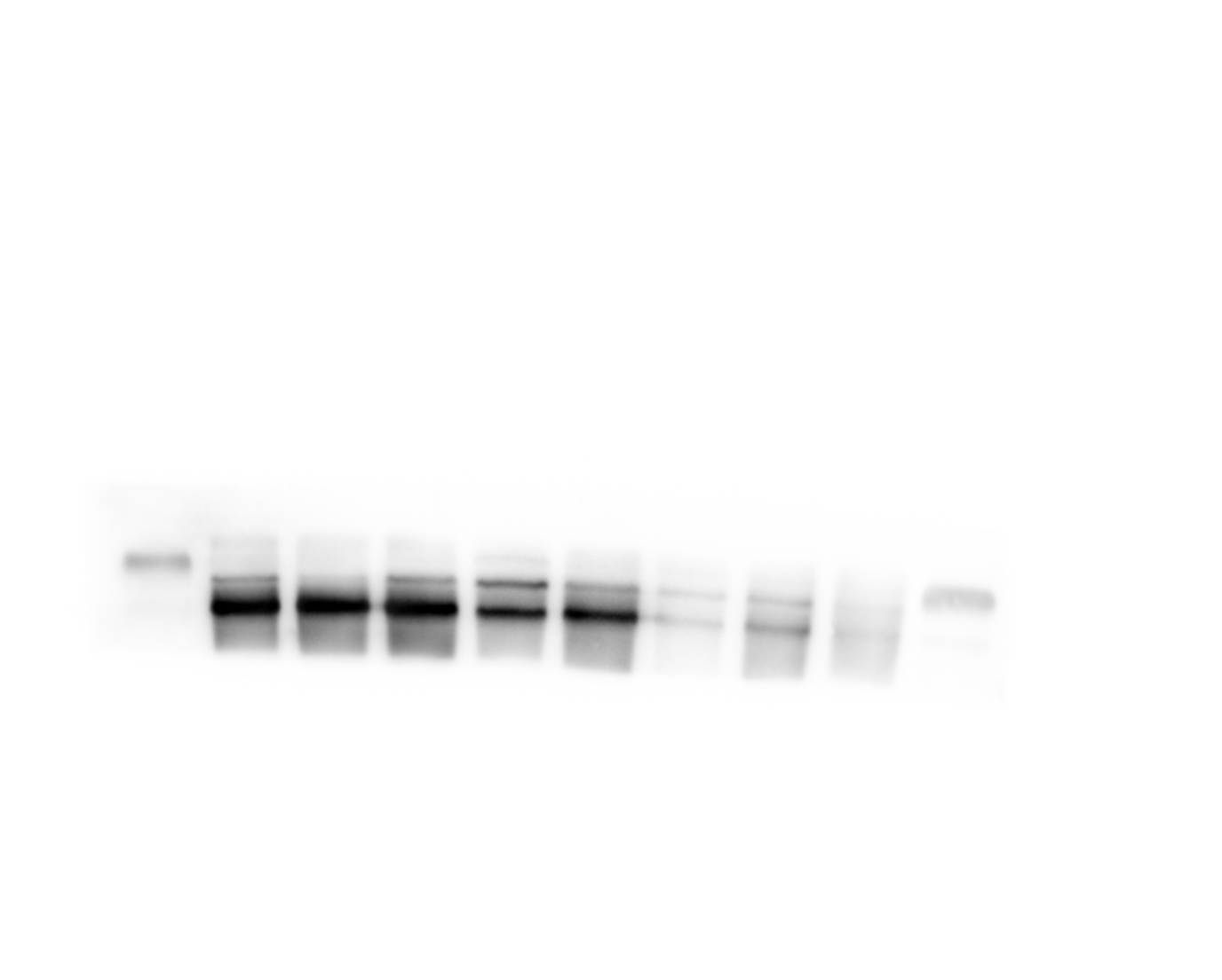

Supplement: Figure 1—source data 1. [file elife-97827-fig1-data1.zip › Figure 1-source data 1.3 .tiff]

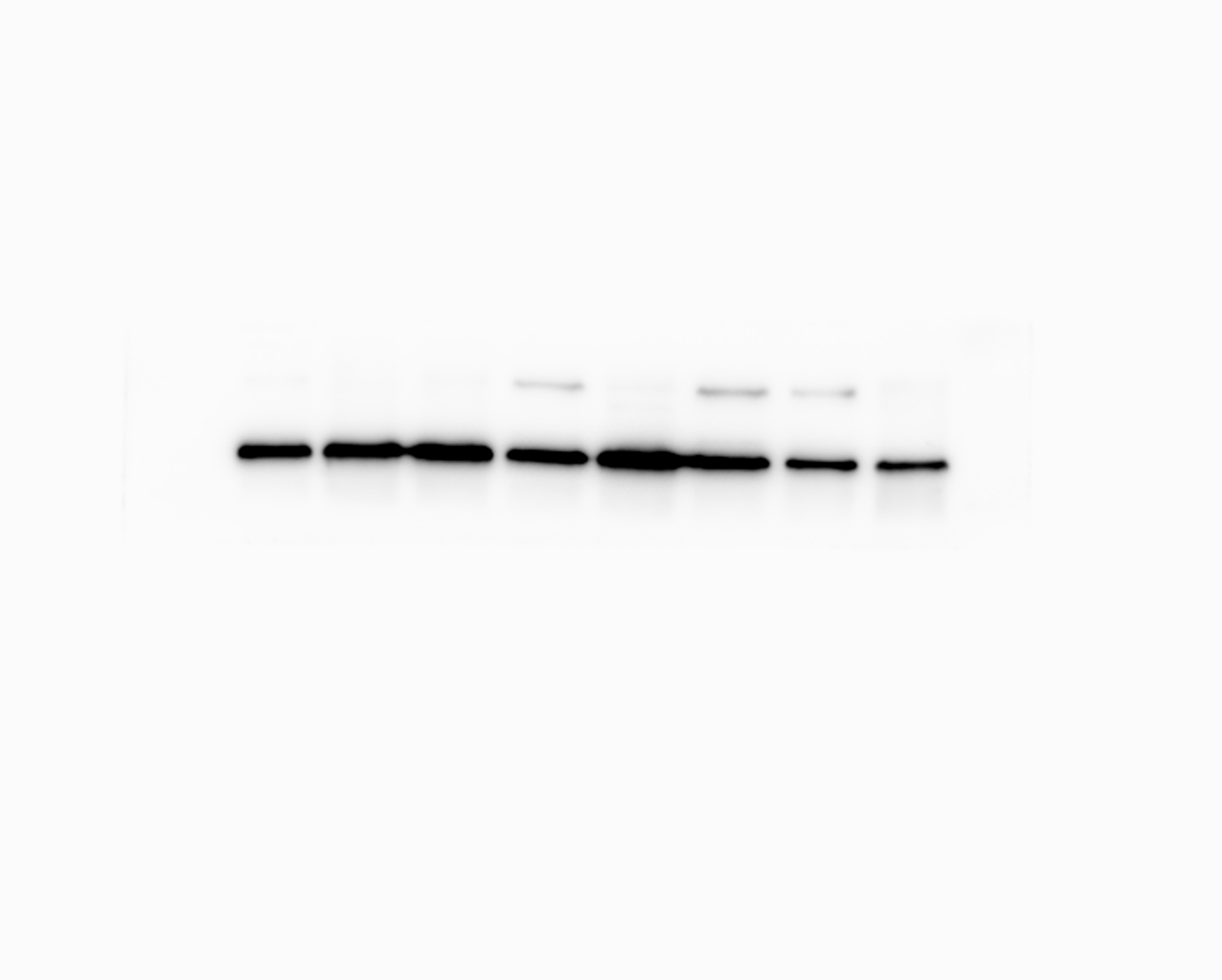

Supplement: Figure 1—source data 1. [file elife-97827-fig1-data1.zip › Figure 1-source data 1.4 .tiff]

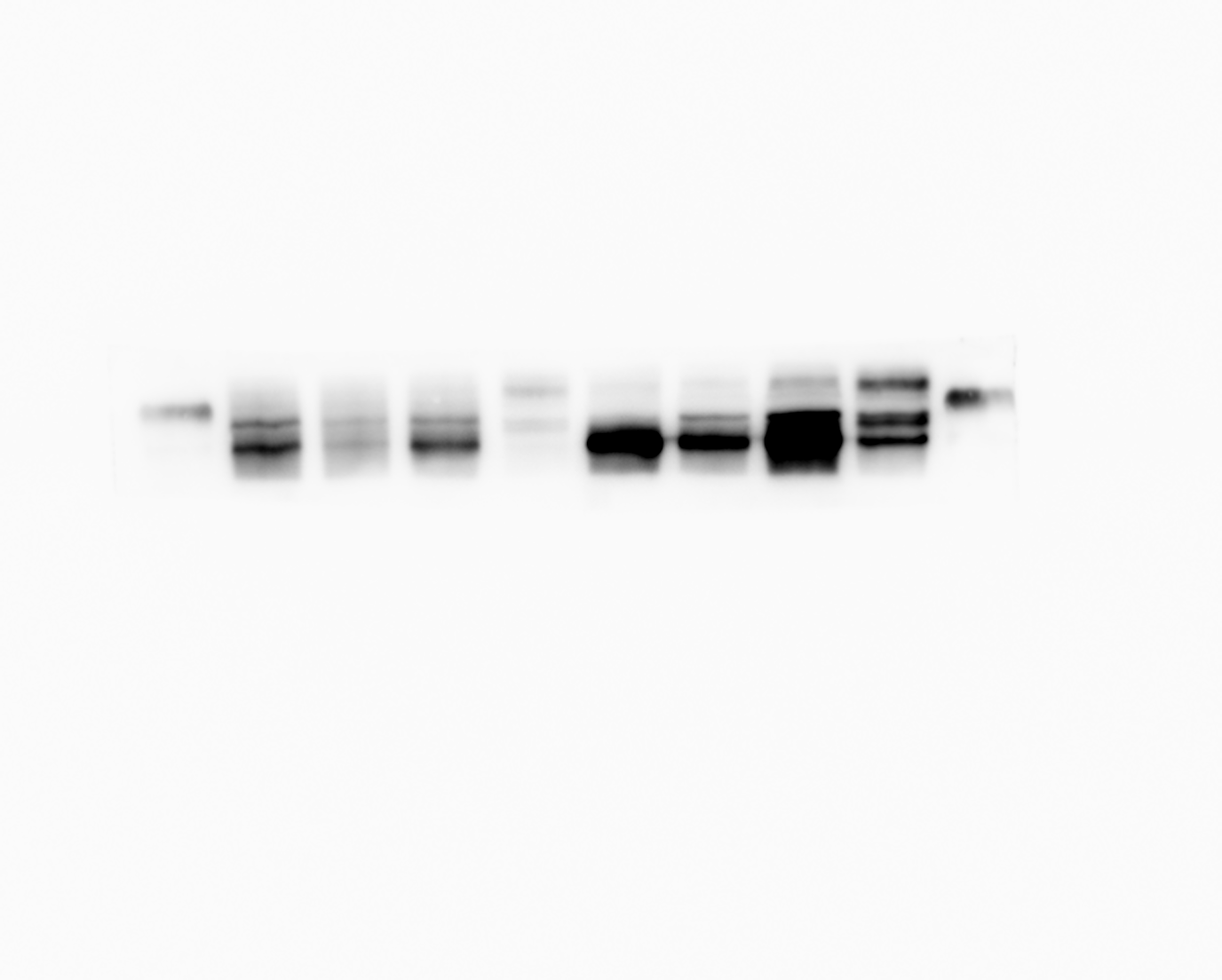

Supplement: Figure 1—source data 1. [file elife-97827-fig1-data1.zip › Figure 1-source data 1.5 .tiff]

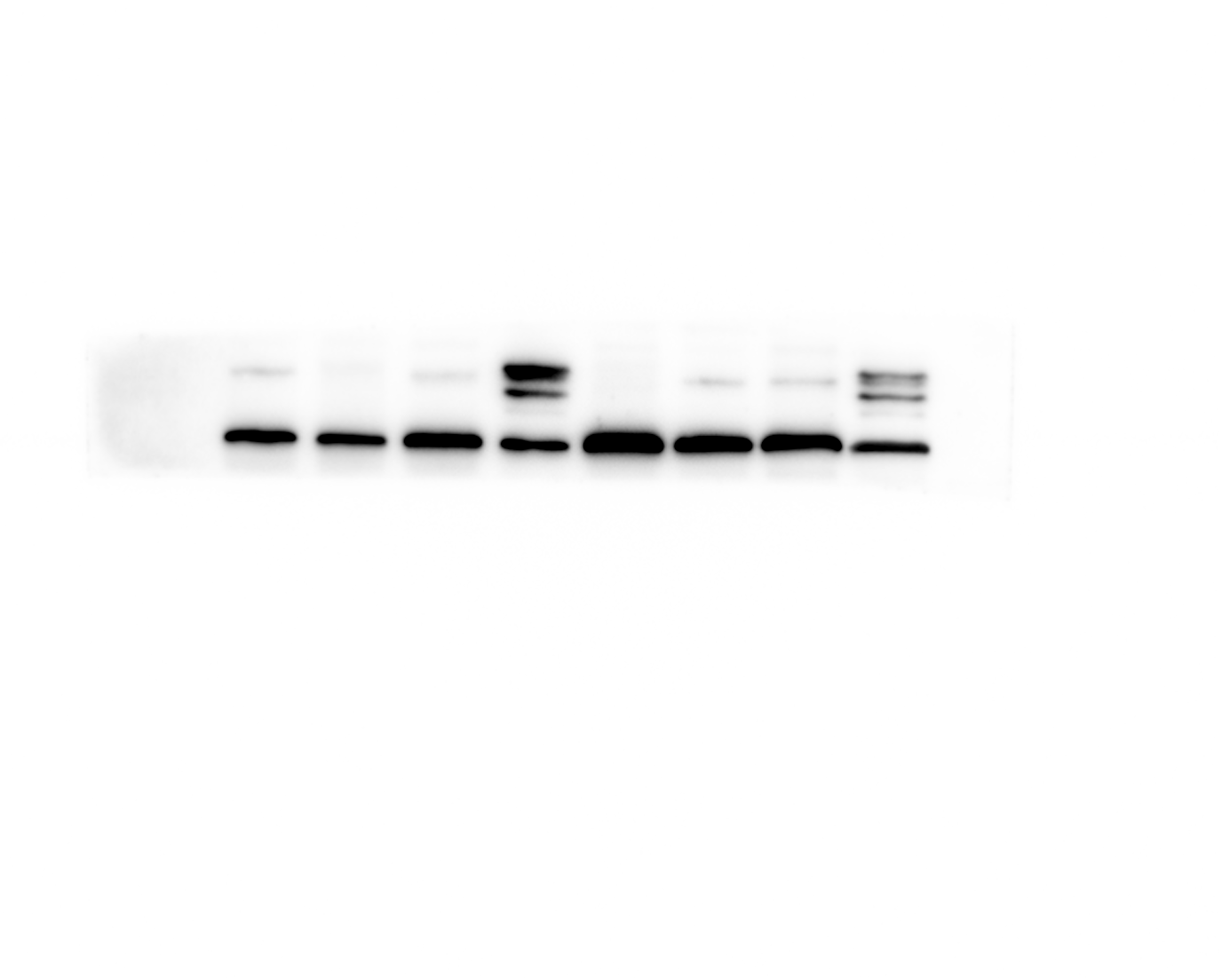

Supplement: Figure 1—source data 1. [file elife-97827-fig1-data1.zip › Figure 1-source data 1.6 .tiff]

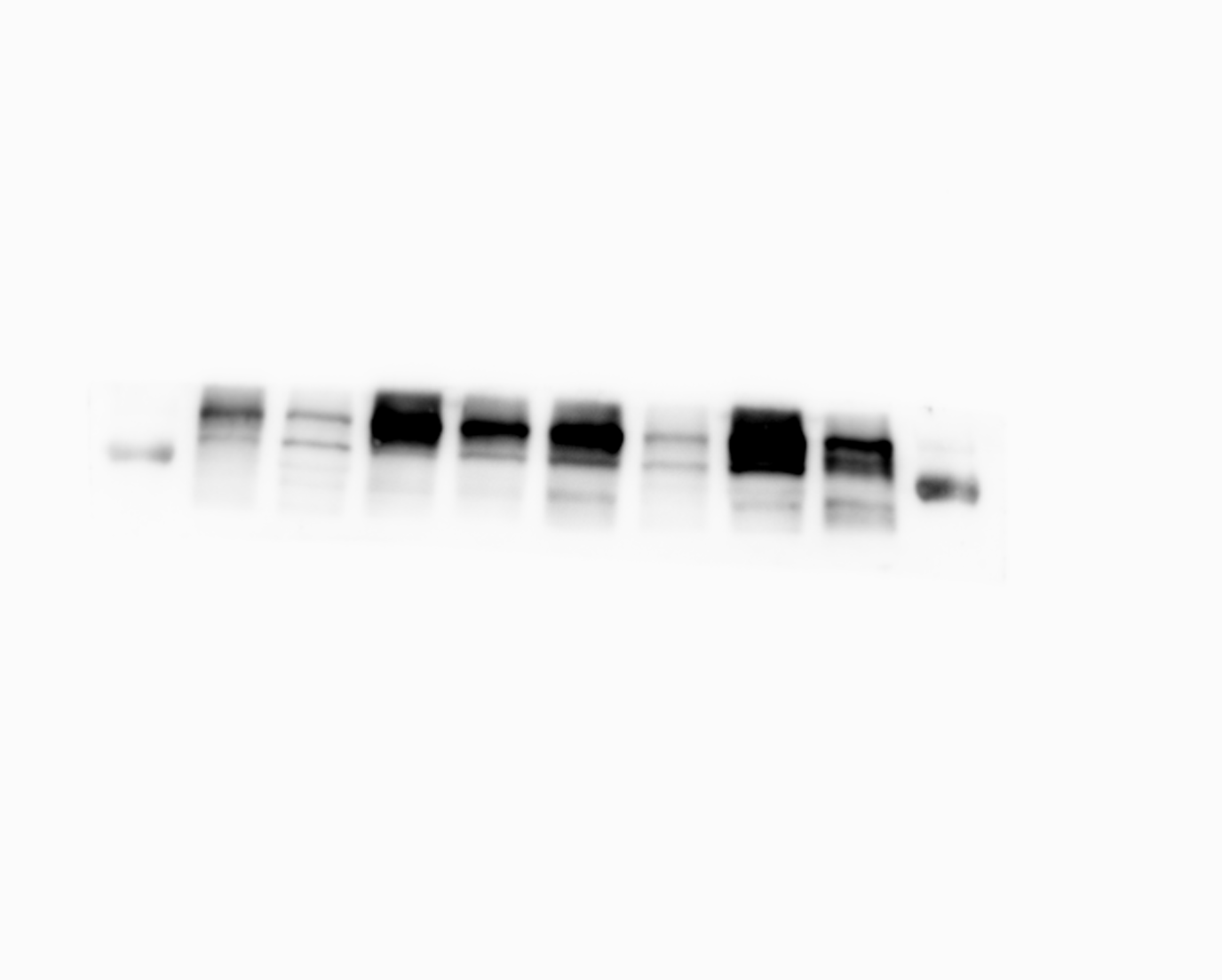

Supplement: Figure 1—source data 1. [file elife-97827-fig1-data1.zip › Figure 1-source data 1.7 .tiff]

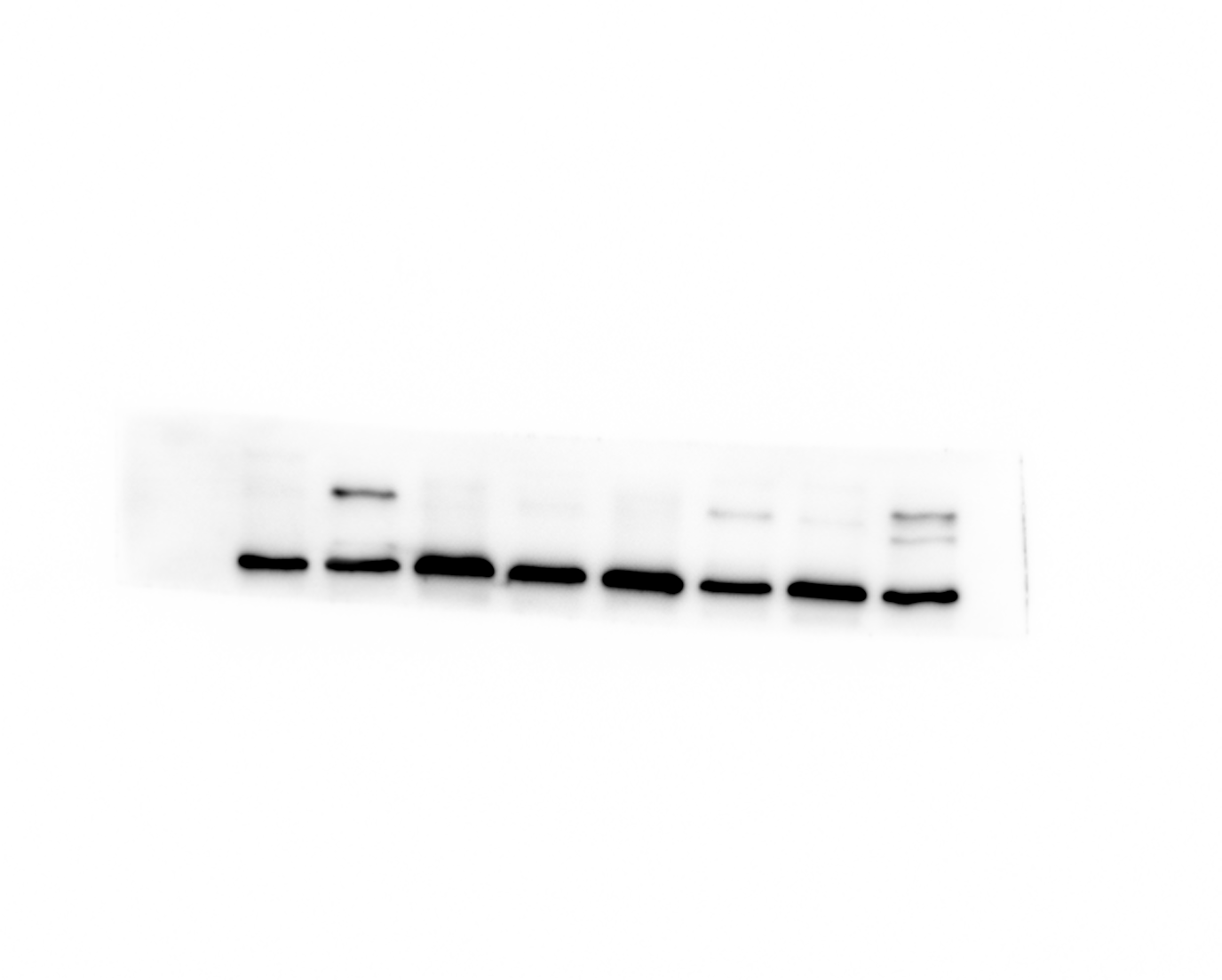

Supplement: Figure 1—source data 1. [file elife-97827-fig1-data1.zip › Figure 1-source data 1.8 .tiff]

### Figure 1C

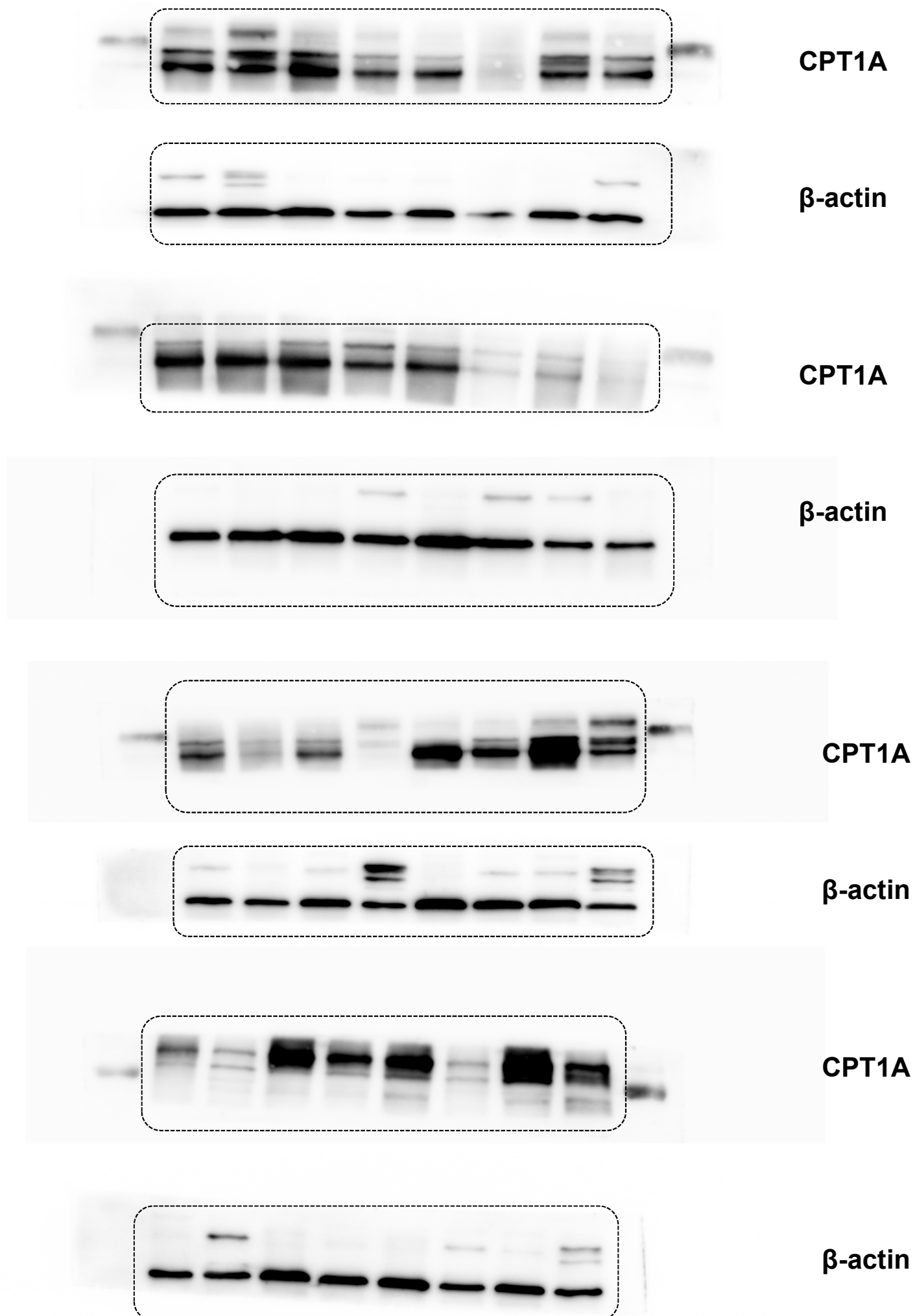

Supplement: Figure 1—source data 2. [file elife-97827-fig1-data2.zip › Figure 1-source data 1.pdf]

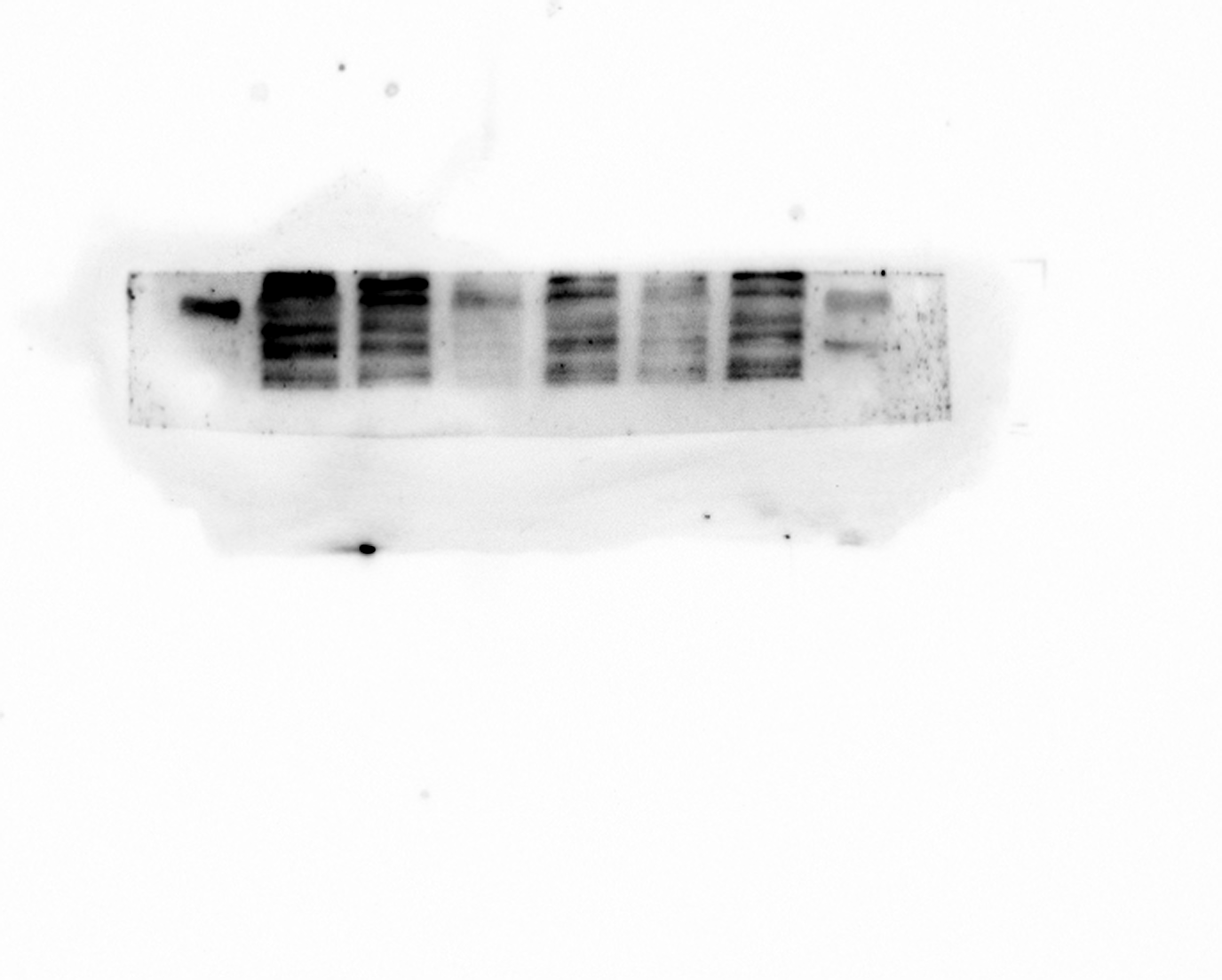

Supplement: Figure 3—source data 1. [file elife-97827-fig3-data1.zip › Figure 3-source data 1.1 .tiff]

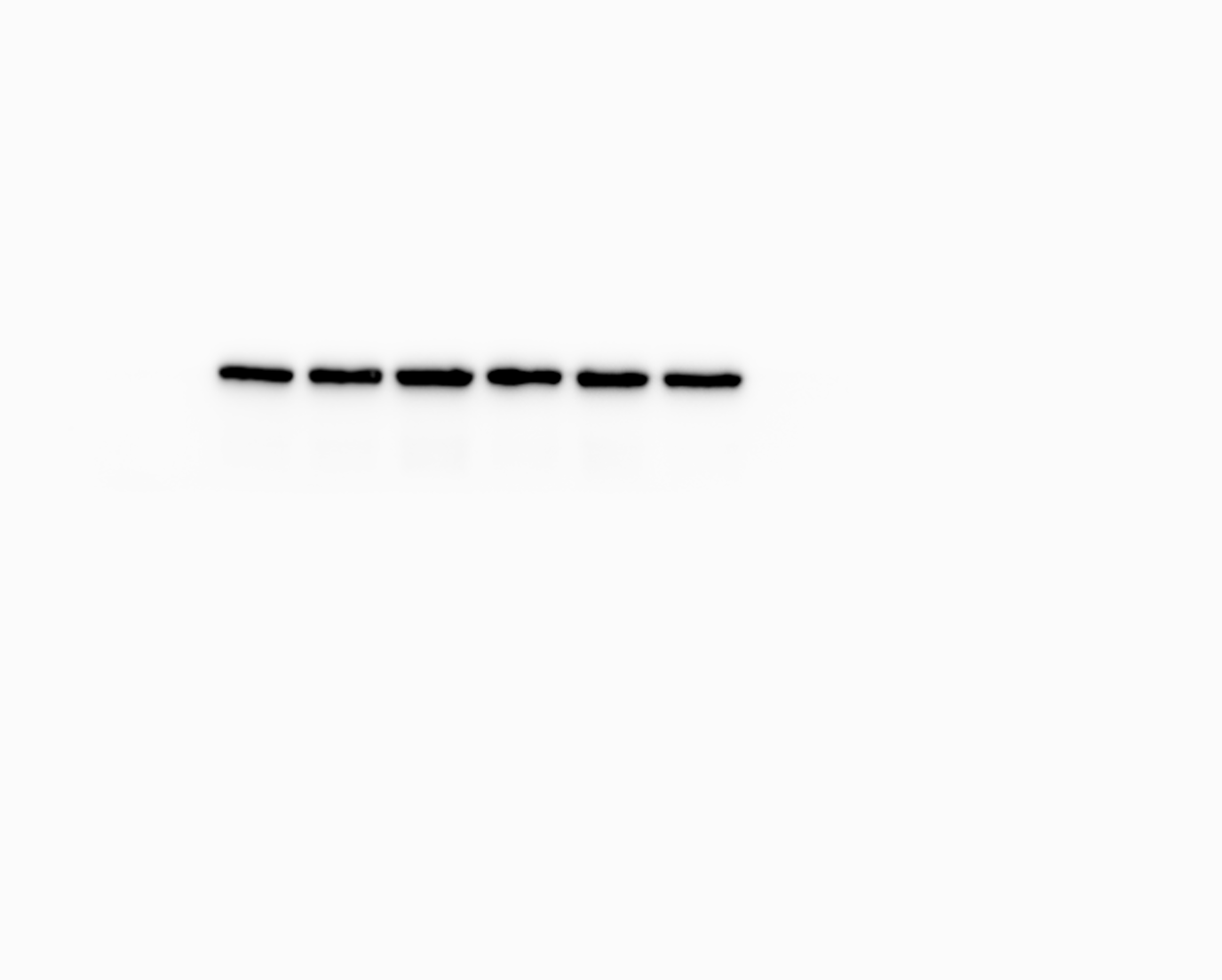

Supplement: Figure 3—source data 1. [file elife-97827-fig3-data1.zip › Figure 3-source data 1.2 .tiff]

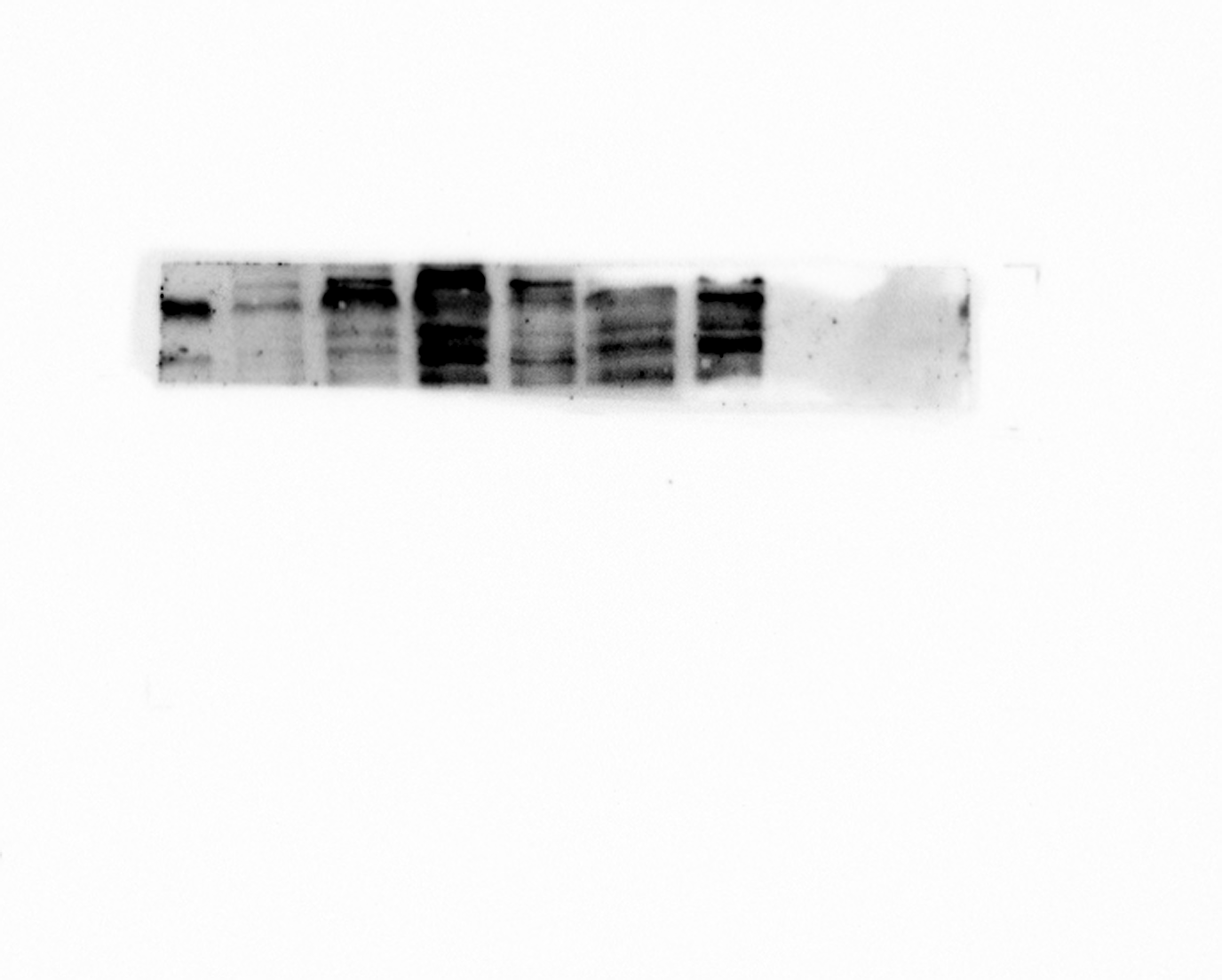

Supplement: Figure 3—source data 1. [file elife-97827-fig3-data1.zip › Figure 3-source data 1.3 .tiff]

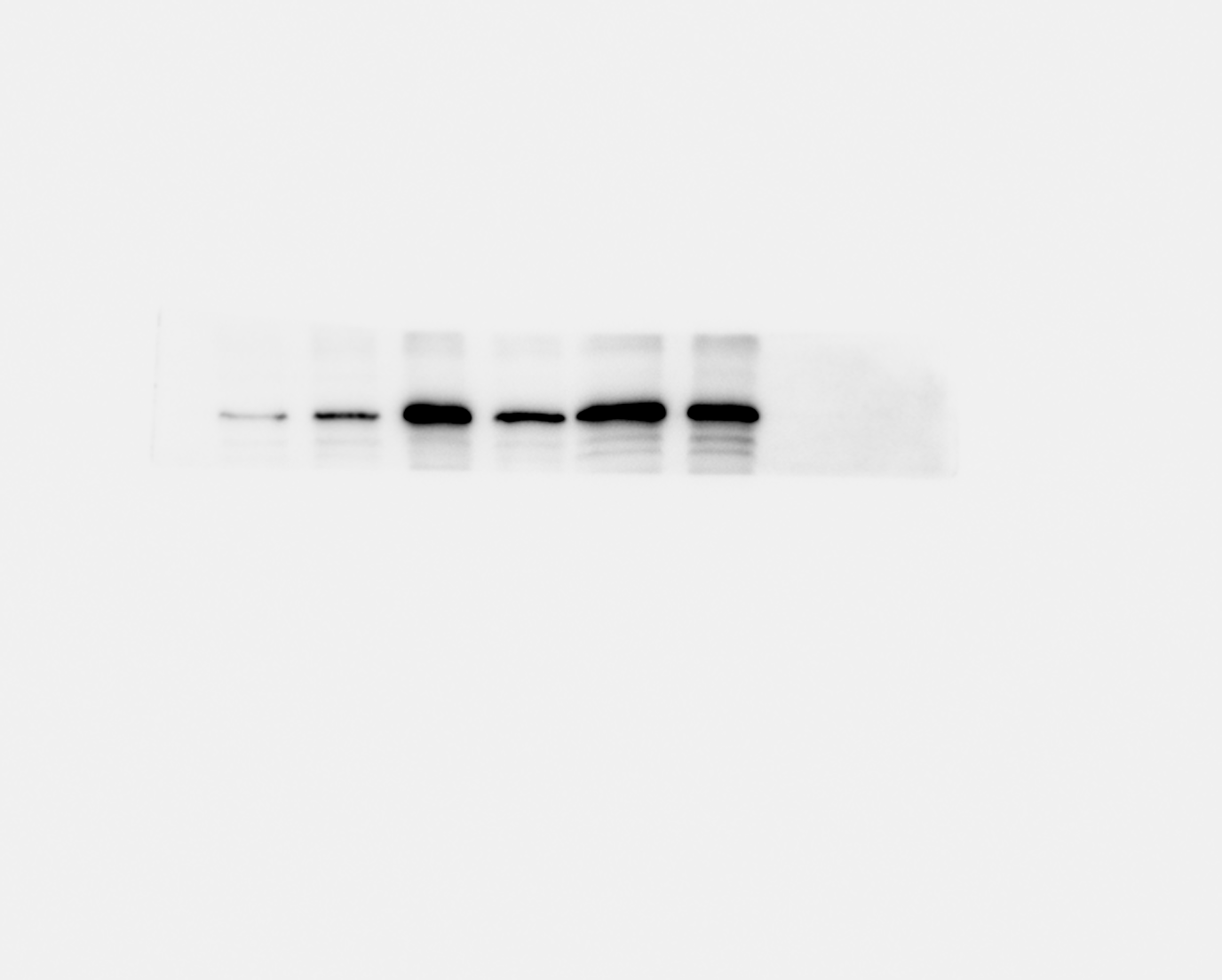

Supplement: Figure 3—source data 1. [file elife-97827-fig3-data1.zip › Figure 3-source data 1.4 .tiff]

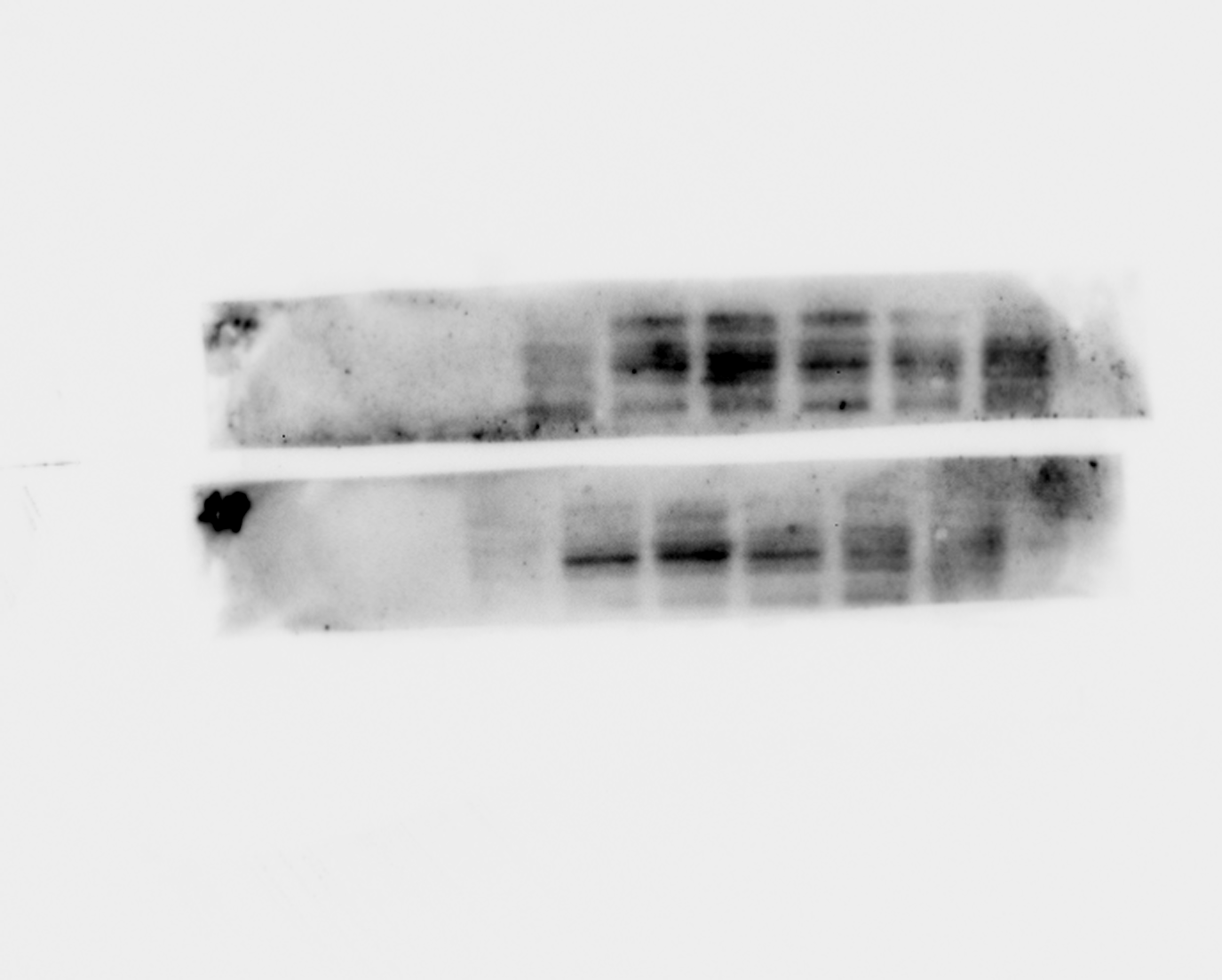

Supplement: Figure 3—source data 1. [file elife-97827-fig3-data1.zip › Figure 3-source data 2.1 .tiff]

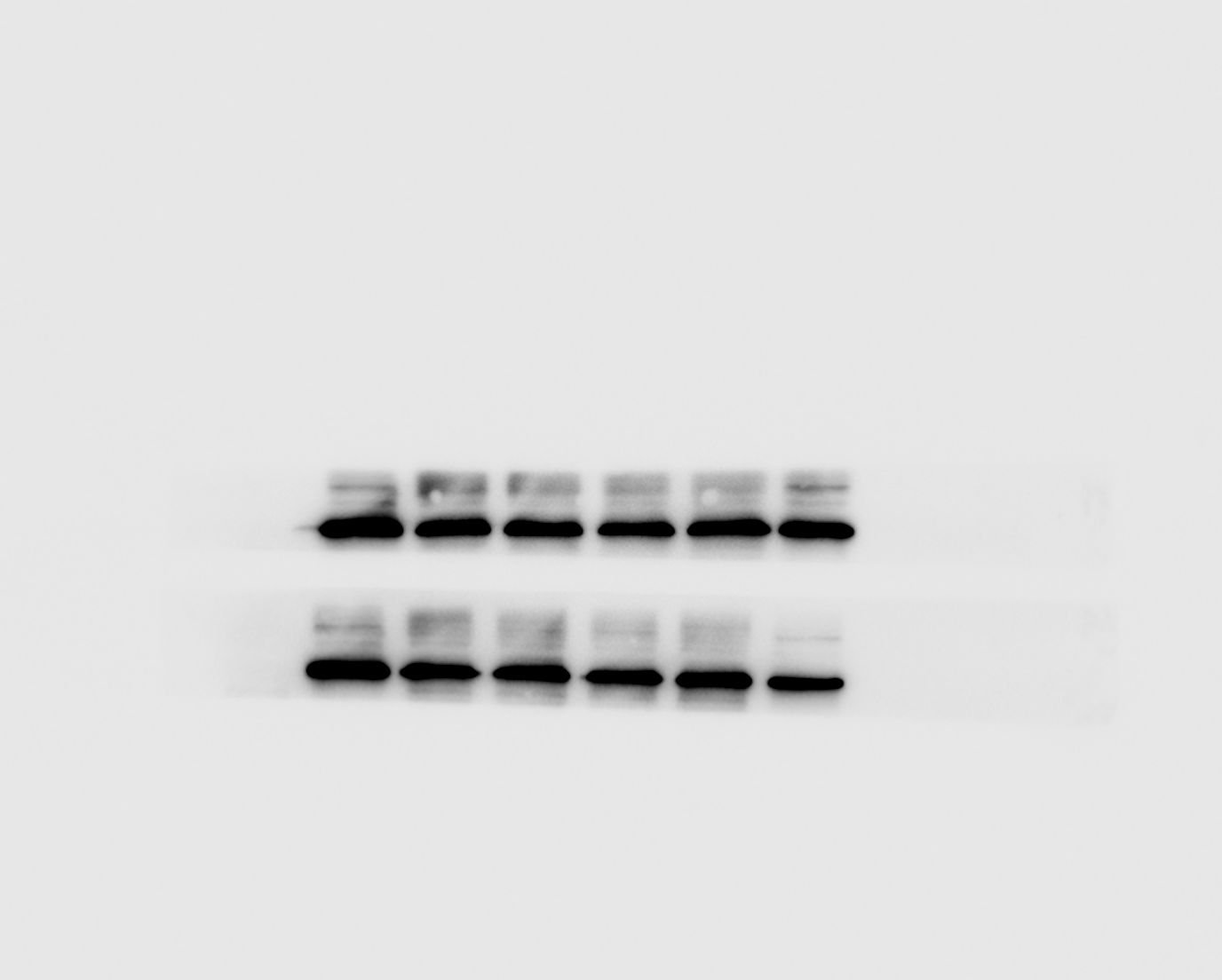

Supplement: Figure 3—source data 1. [file elife-97827-fig3-data1.zip › Figure 3-source data 2.2 .tiff]

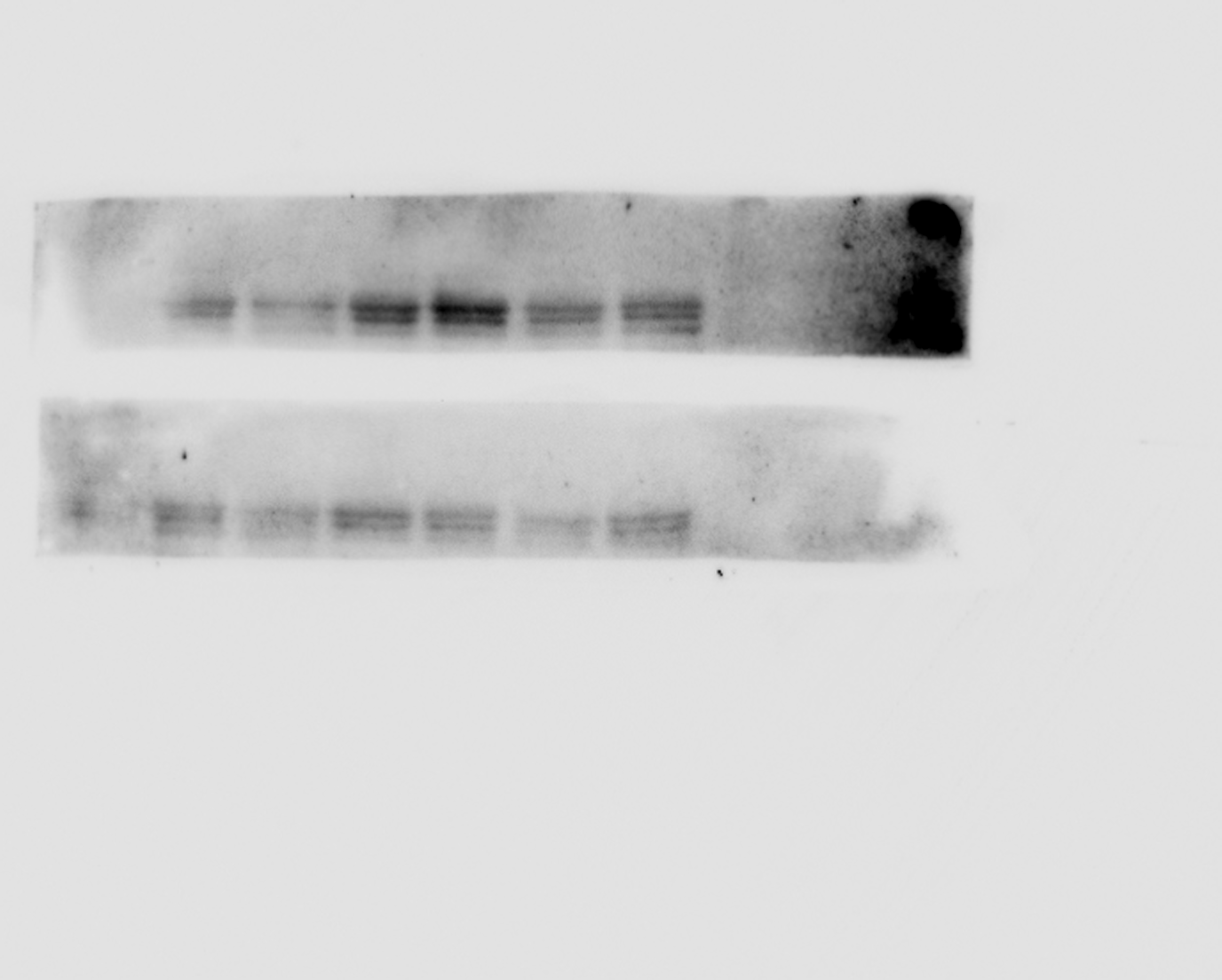

Supplement: Figure 3—source data 1. [file elife-97827-fig3-data1.zip › Figure 3-source data 3.1 .tiff]

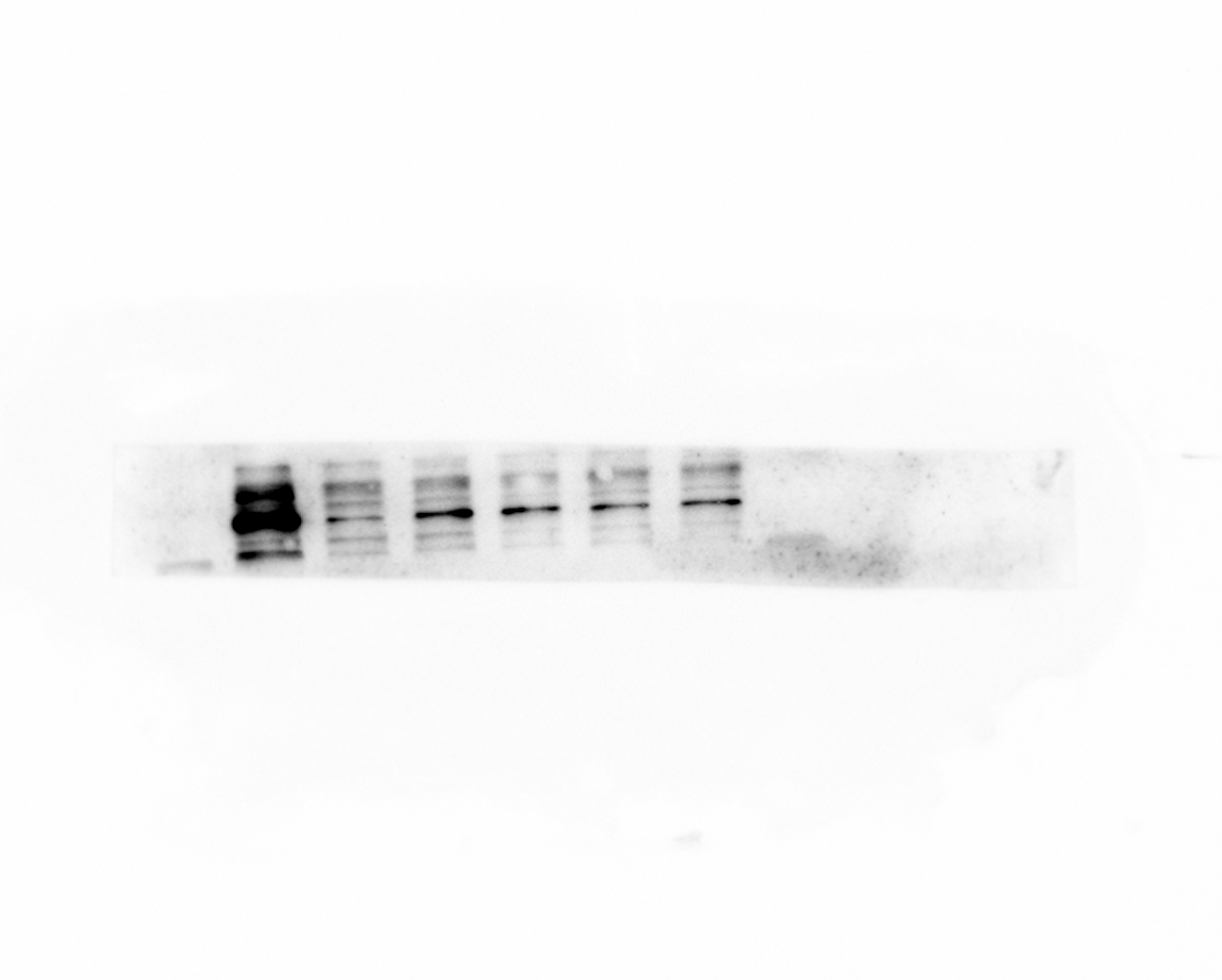

Supplement: Figure 3—source data 1. [file elife-97827-fig3-data1.zip › Figure 3-source data 3.2 .tiff]

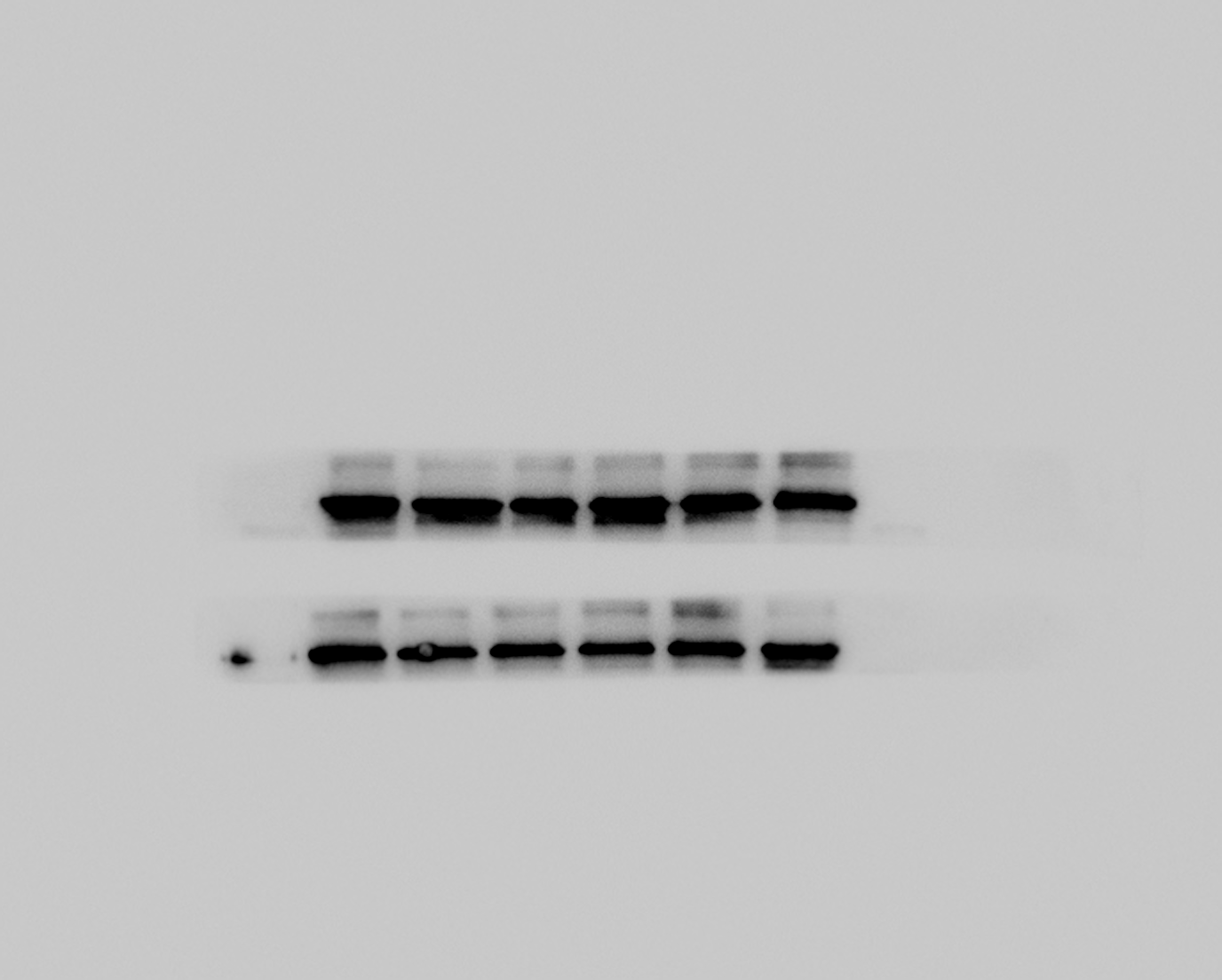

Supplement: Figure 3—source data 1. [file elife-97827-fig3-data1.zip › Figure 3-source data 3.3 .tiff]

Figure 3A

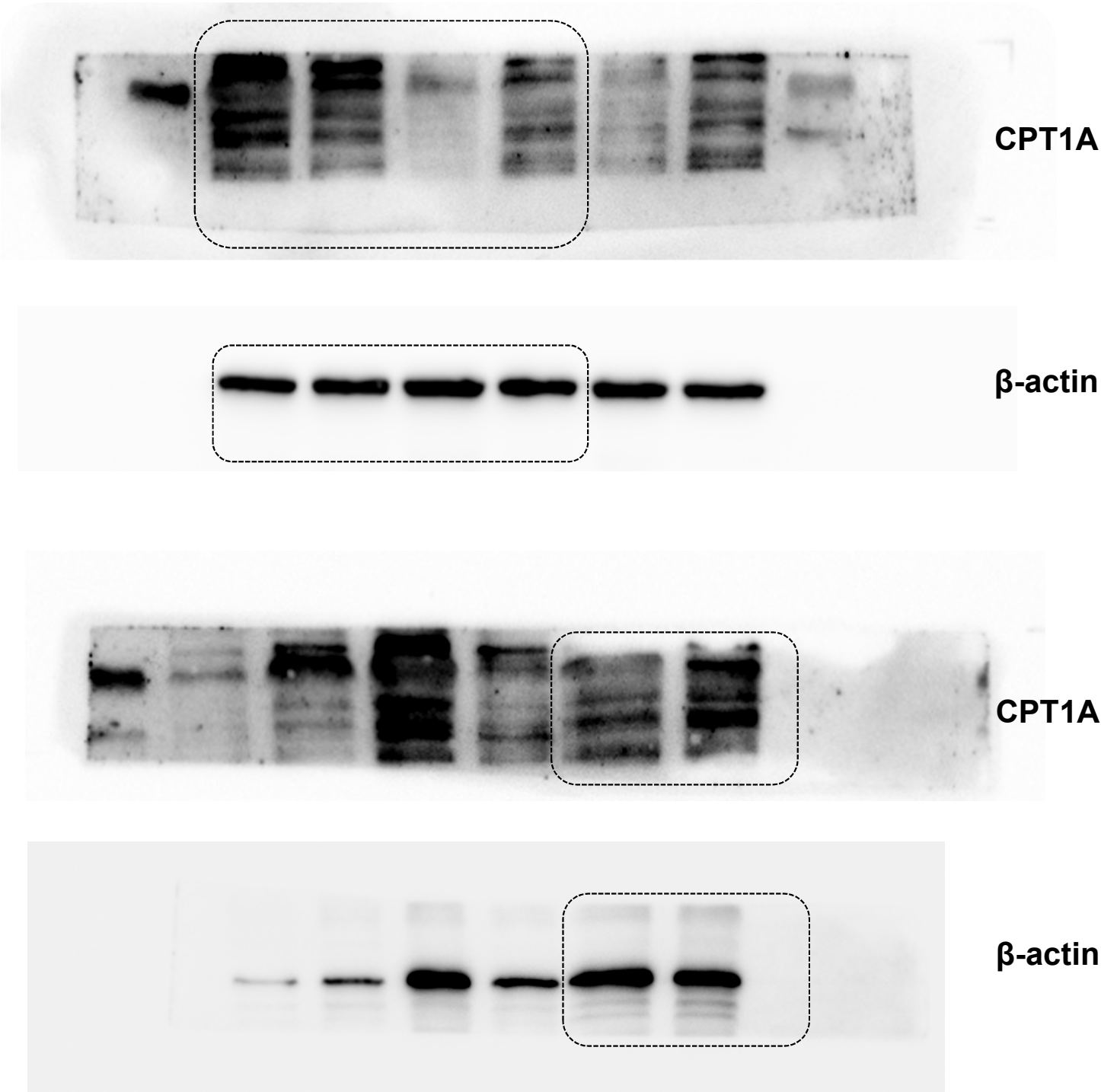

Supplement: Figure 3—source data 2. [file elife-97827-fig3-data2.zip › Figure 3-source data 1.pdf]

Figure 3H

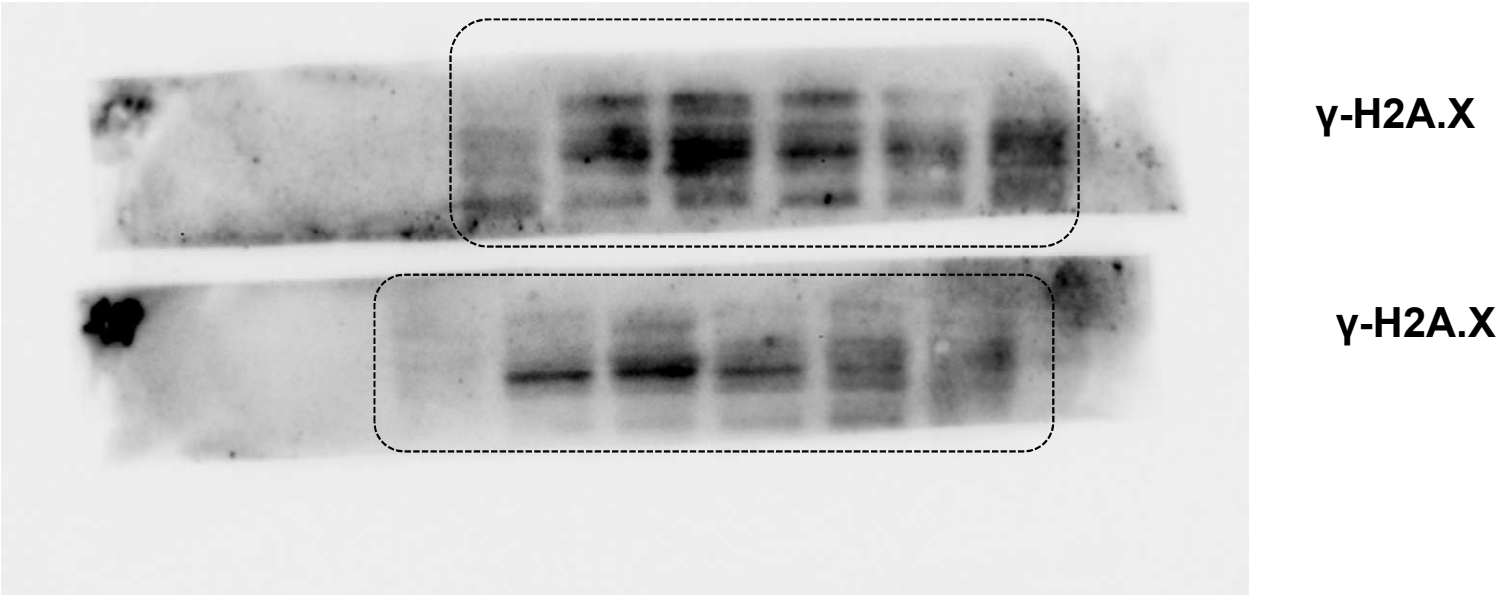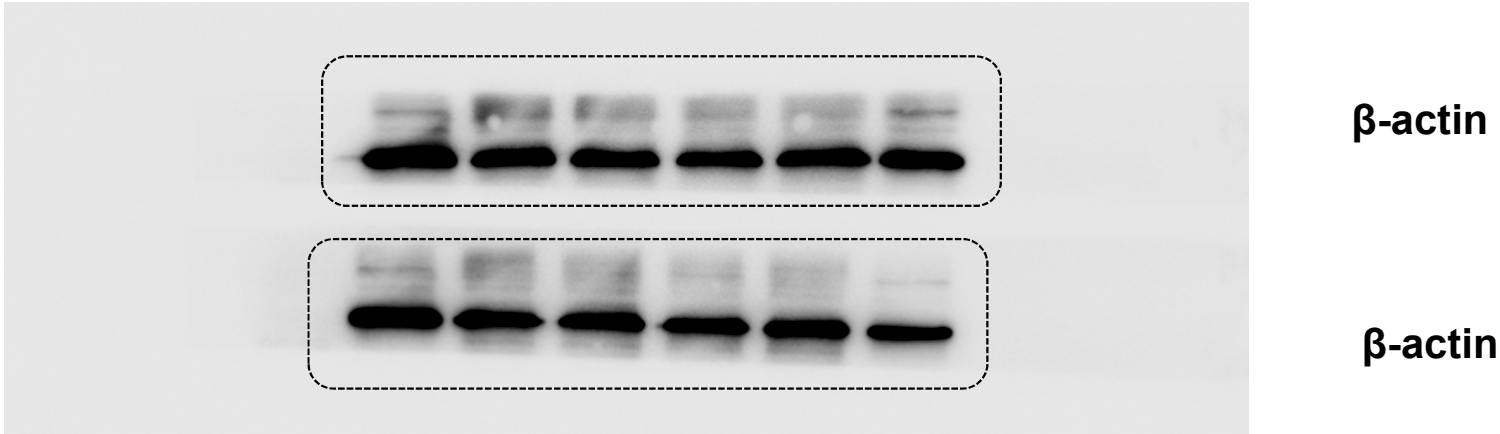

Supplement: Figure 3—source data 2. [file elife-97827-fig3-data2.zip › Figure 3-source data 2.pdf]

Figure 3l

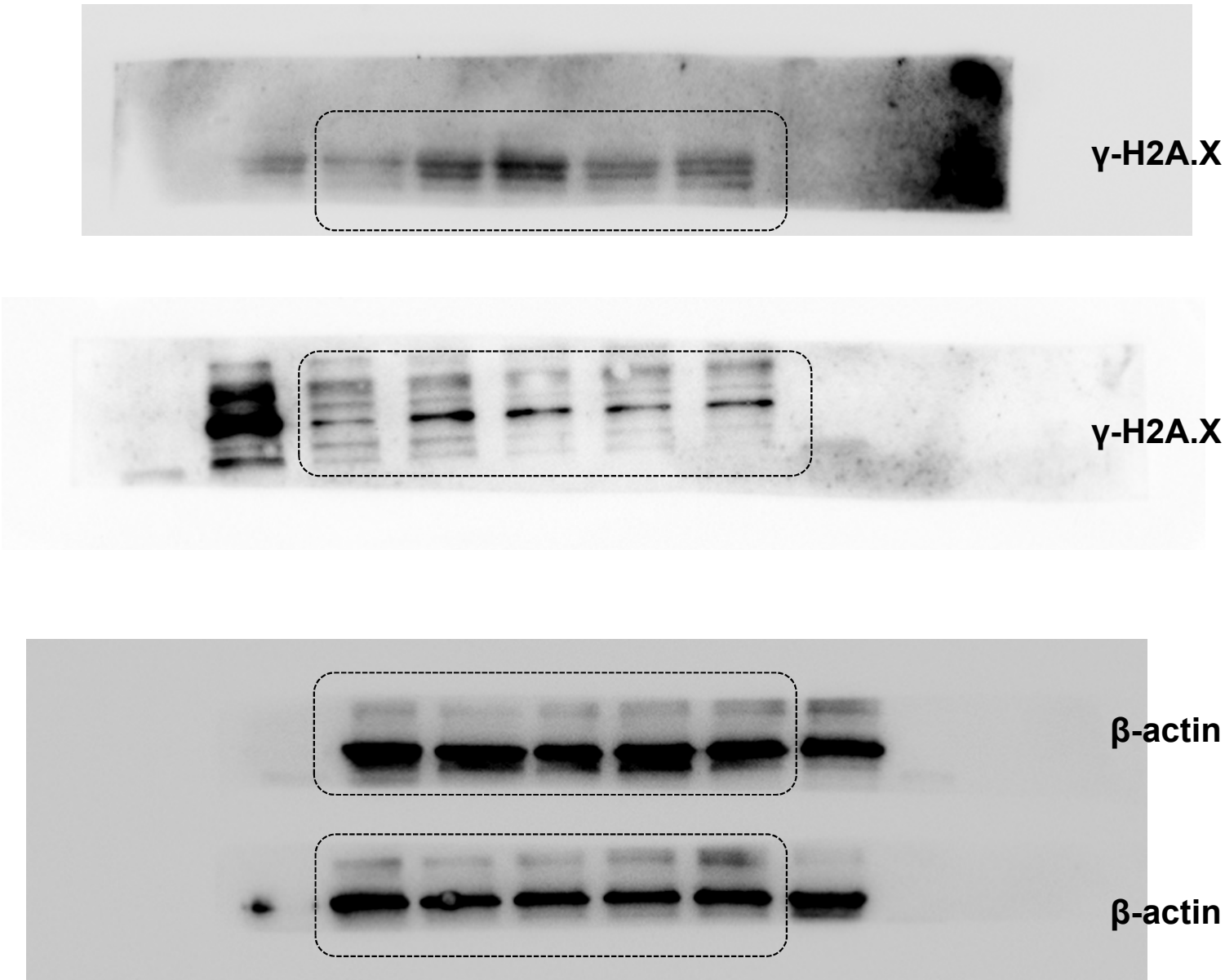

Supplement: Figure 3—source data 2. [file elife-97827-fig3-data2.zip › Figure 3-source data 3.pdf]

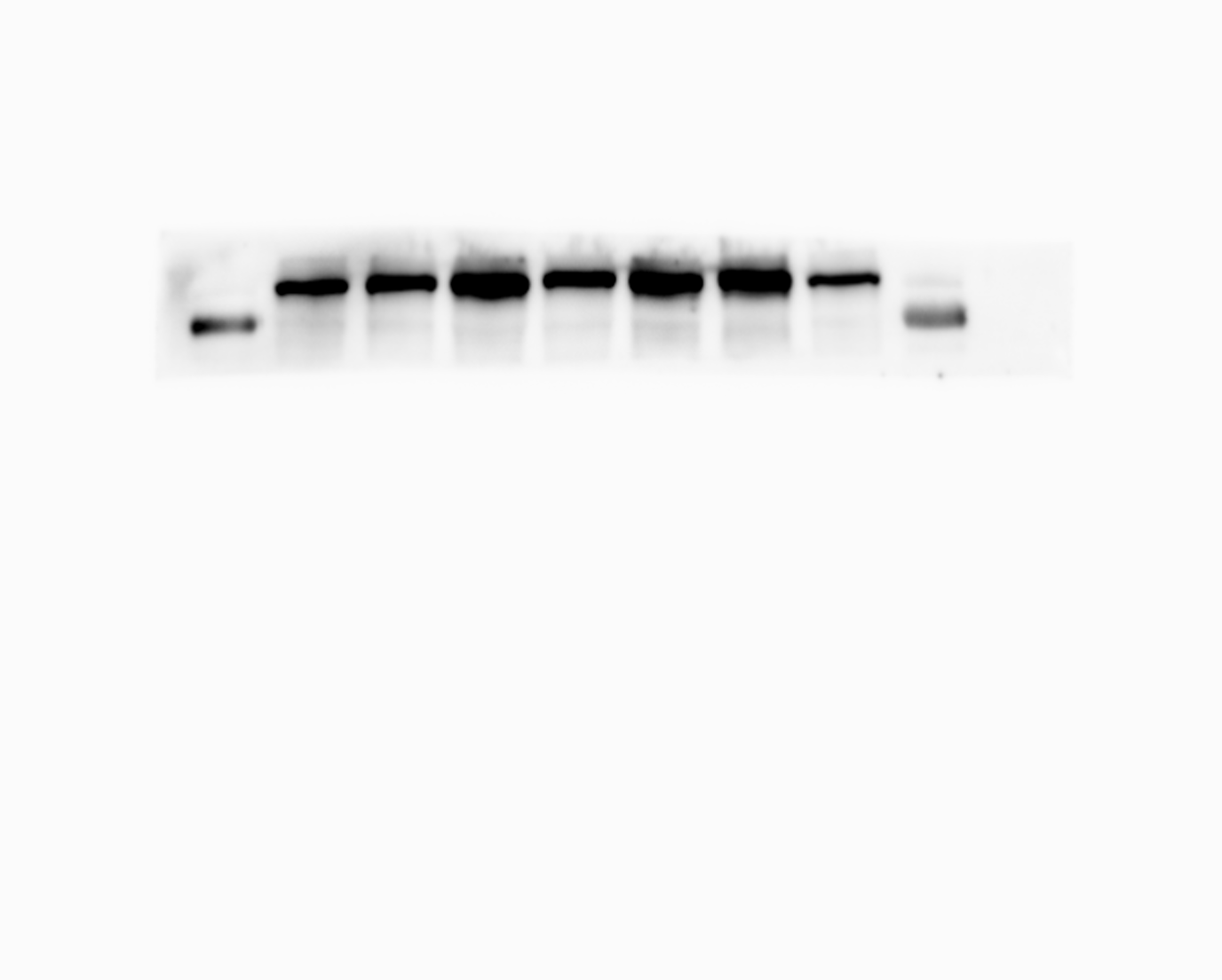

Supplement: Figure 3—figure supplement 1—source data 1. [file elife-97827-fig3-figsupp1-data1.zip › Figure 3-figure supplement 1-source data 1.1 .tiff]

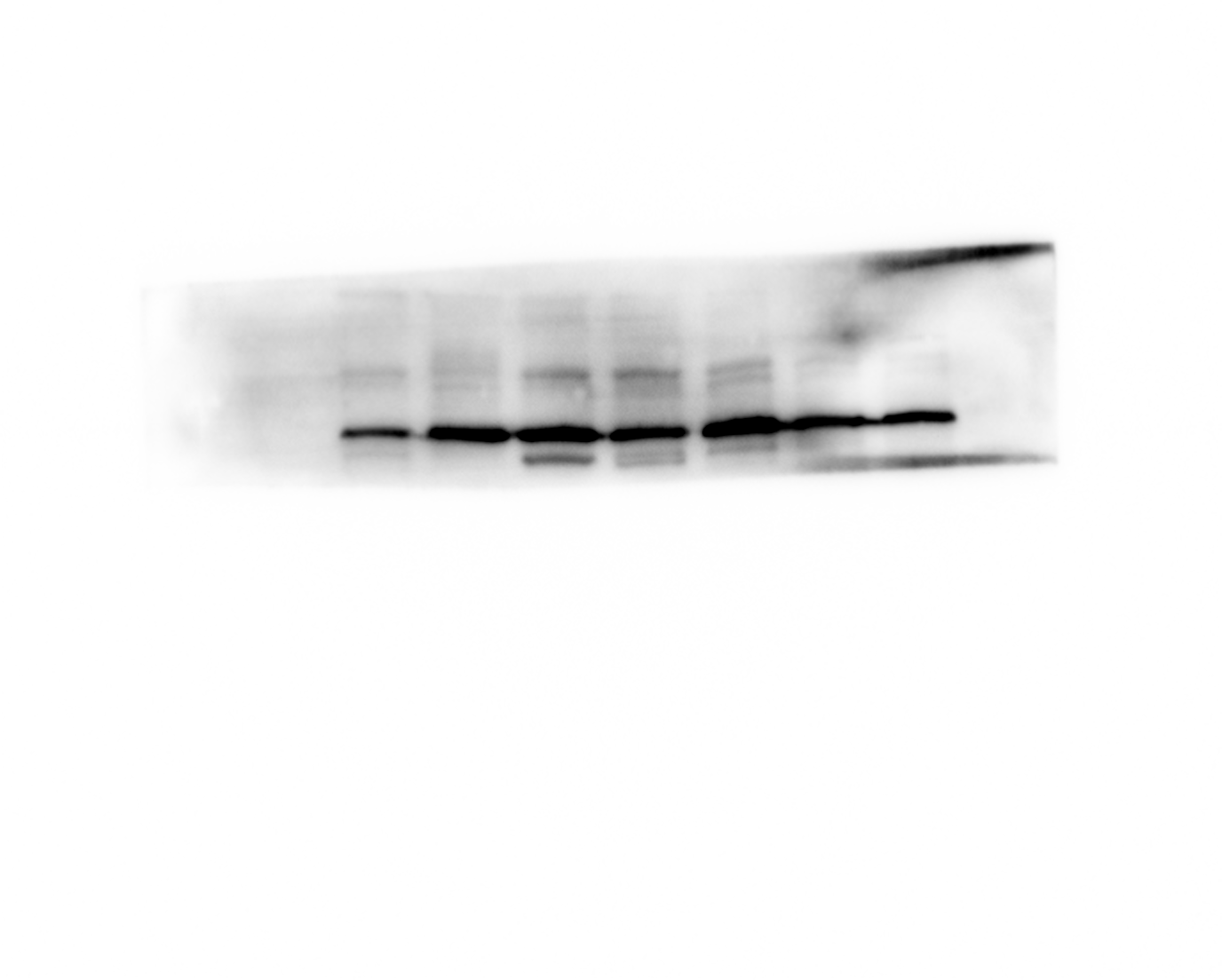

Supplement: Figure 3—figure supplement 1—source data 1. [file elife-97827-fig3-figsupp1-data1.zip › Figure 3-figure supplement 1-source data 1.2 .tiff]

Figure 3-figure supplement 1A

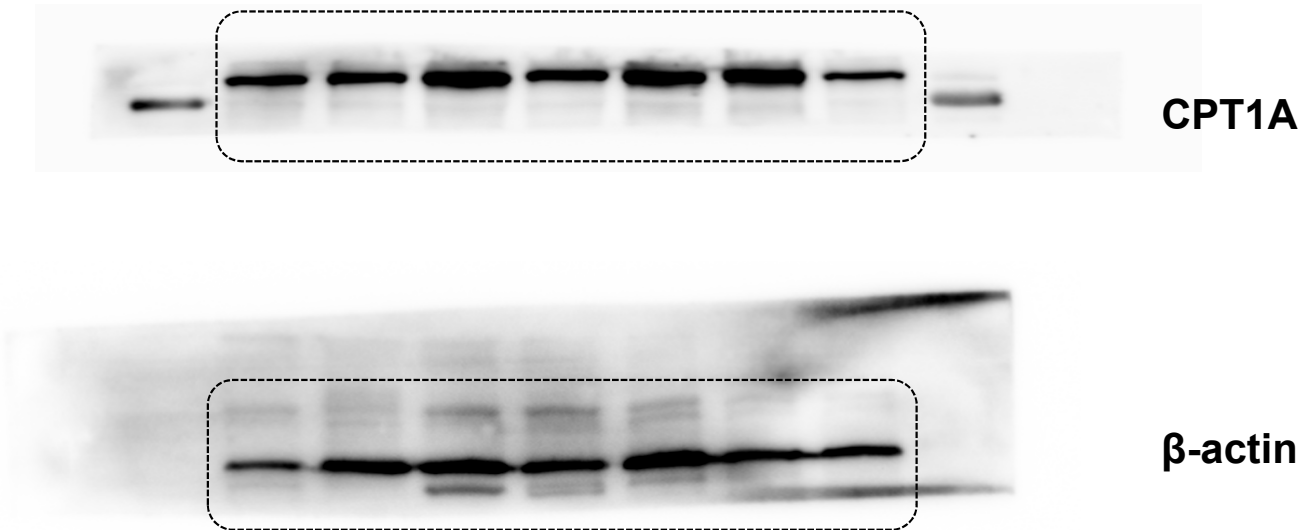

Supplement: Figure 3—figure supplement 1—source data 2. [file elife-97827-fig3-figsupp1-data2.zip › Figure 3-figure supplement 1-source data 1.pdf]

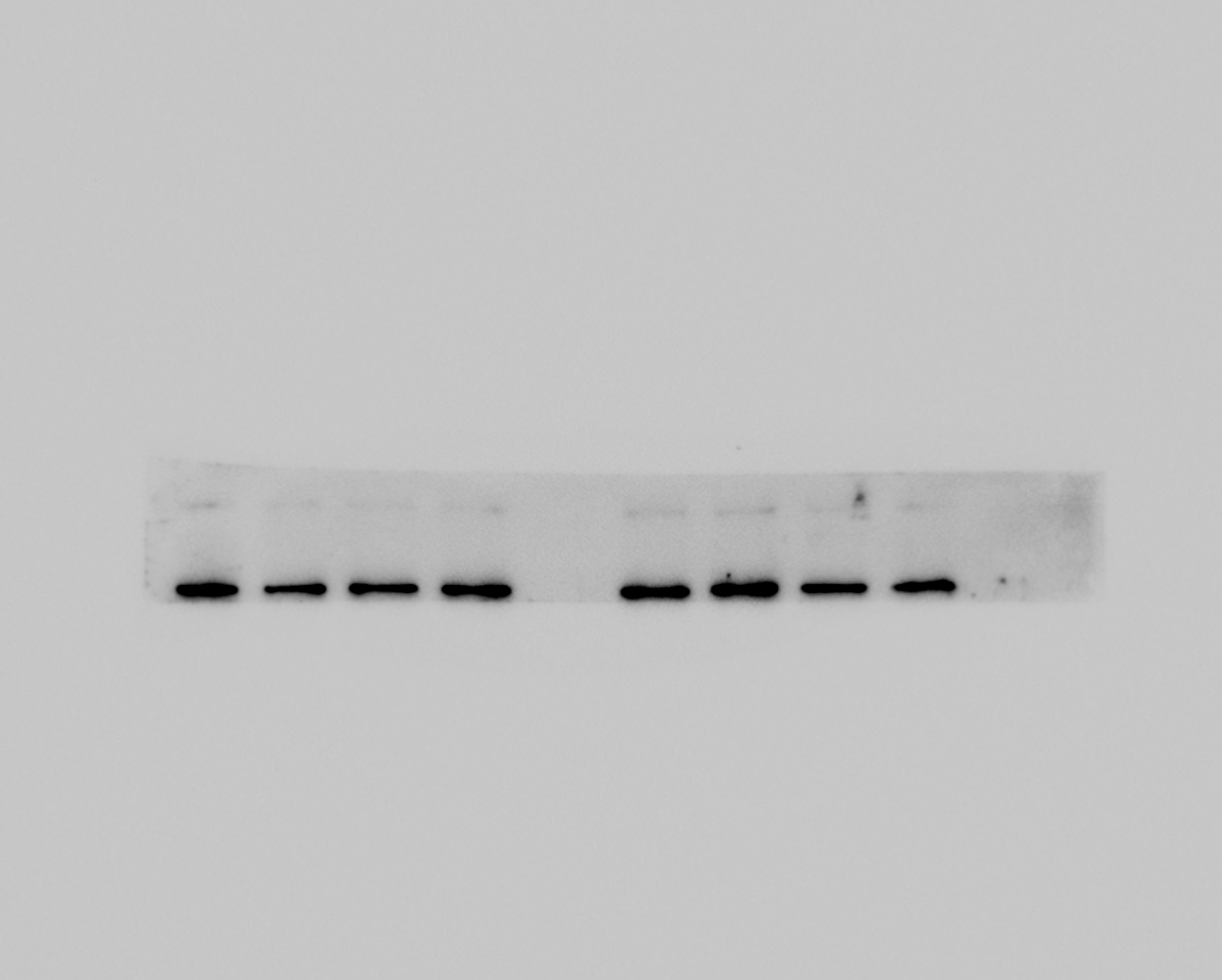

Supplement: Figure 3—figure supplement 2—source data 1. [file elife-97827-fig3-figsupp2-data1.zip › Figure 3-figure supplement 2-source data 1.1 .tiff]

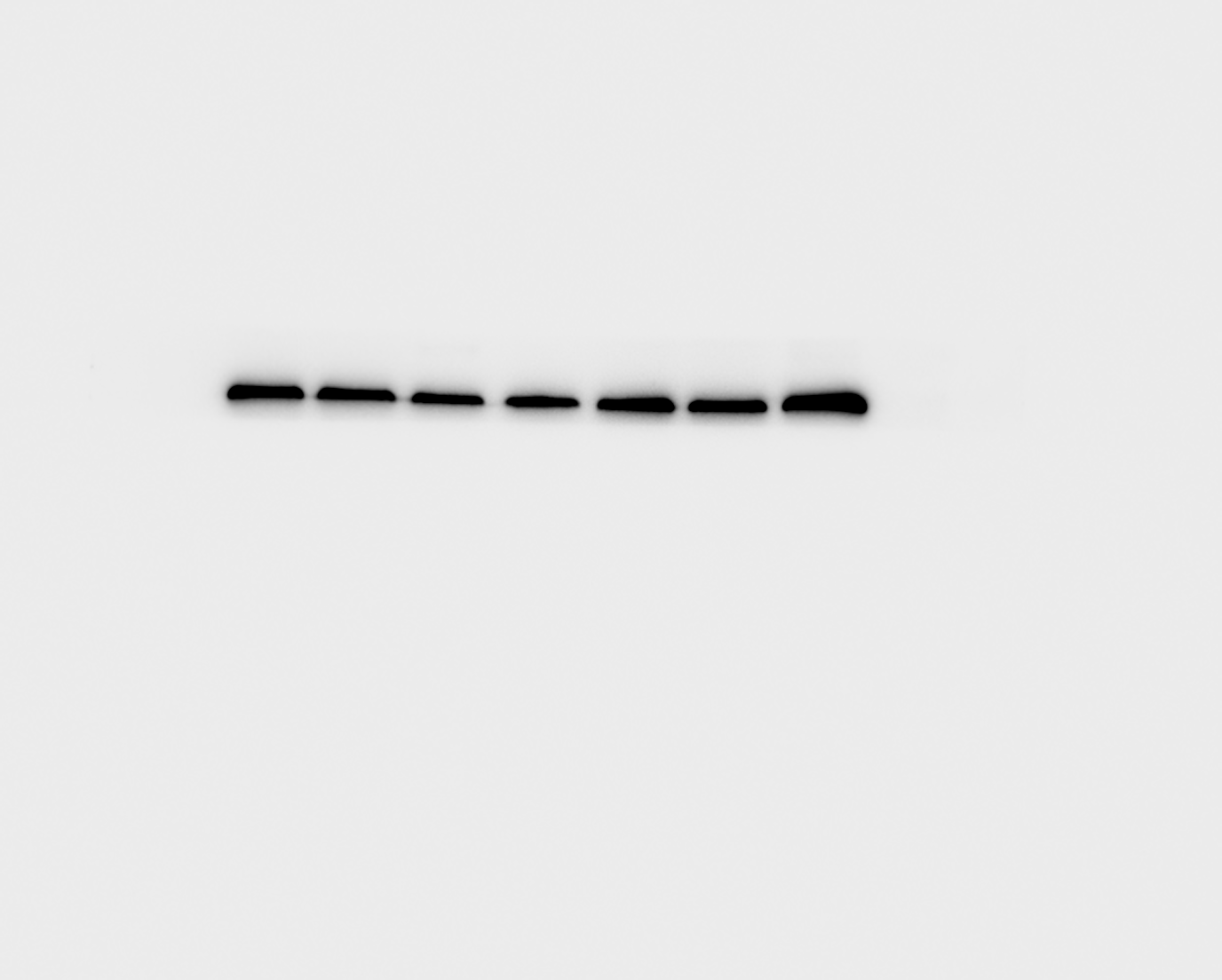

Supplement: Figure 3—figure supplement 2—source data 1. [file elife-97827-fig3-figsupp2-data1.zip › Figure 3-figure supplement 2-source data 1.2 .tiff]

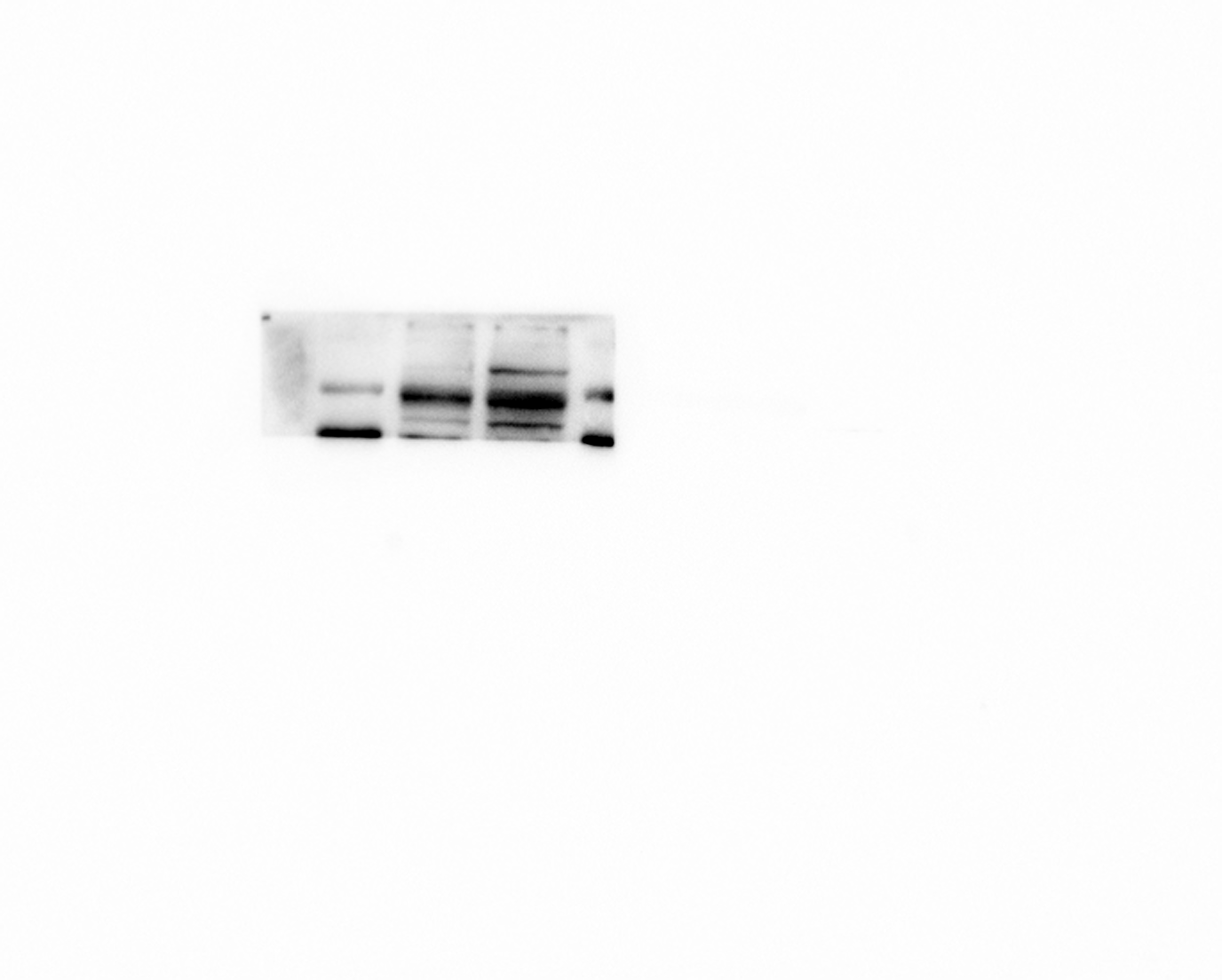

Supplement: Figure 3—figure supplement 2—source data 1. [file elife-97827-fig3-figsupp2-data1.zip › Figure 3-figure supplement 2-source data 2.1 .tiff]

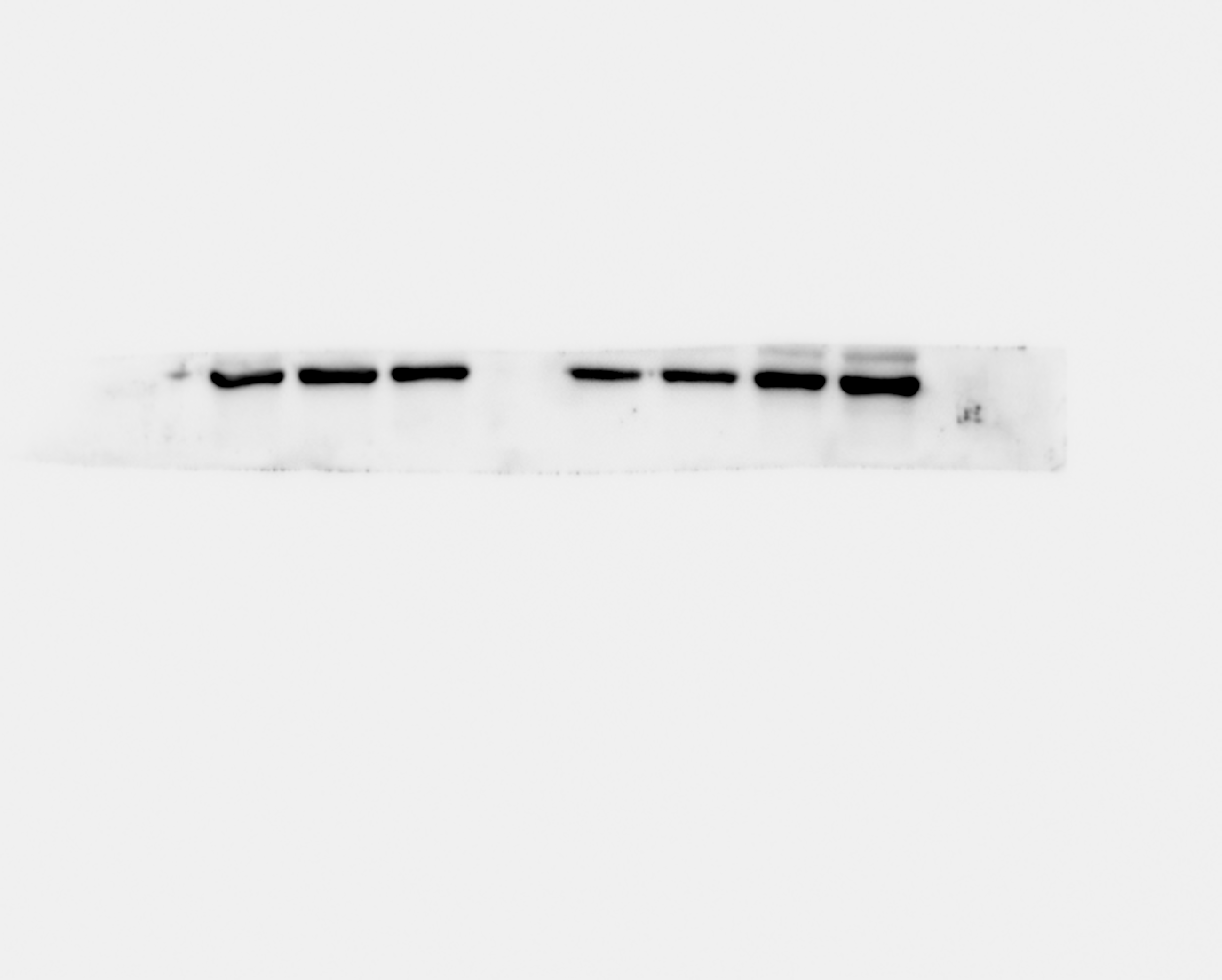

Supplement: Figure 3—figure supplement 2—source data 1. [file elife-97827-fig3-figsupp2-data1.zip › Figure 3-figure supplement 2-source data 2.2 .tiff]

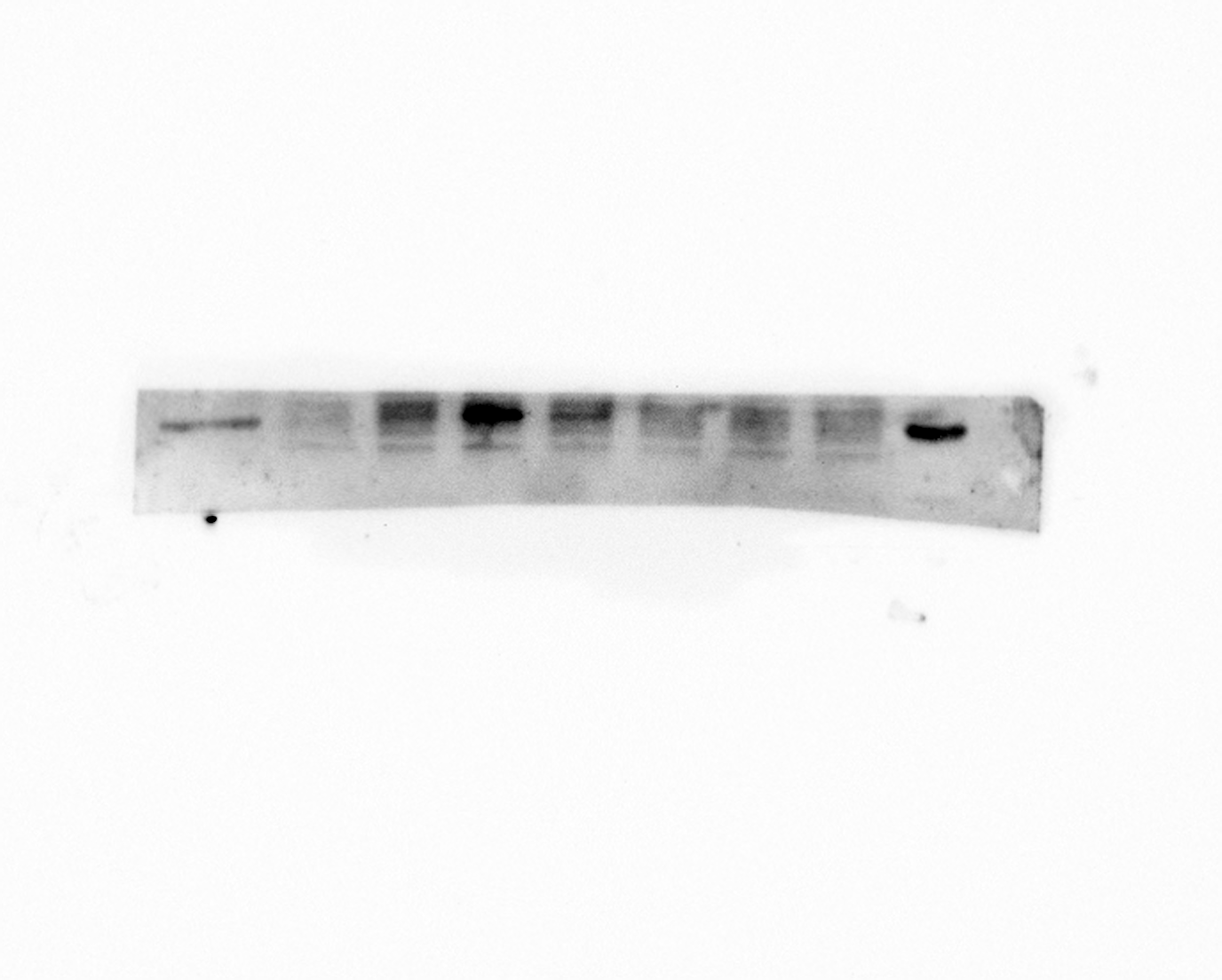

Supplement: Figure 3—figure supplement 2—source data 1. [file elife-97827-fig3-figsupp2-data1.zip › Figure 3-figure supplement 2-source data 3.1 .tiff]

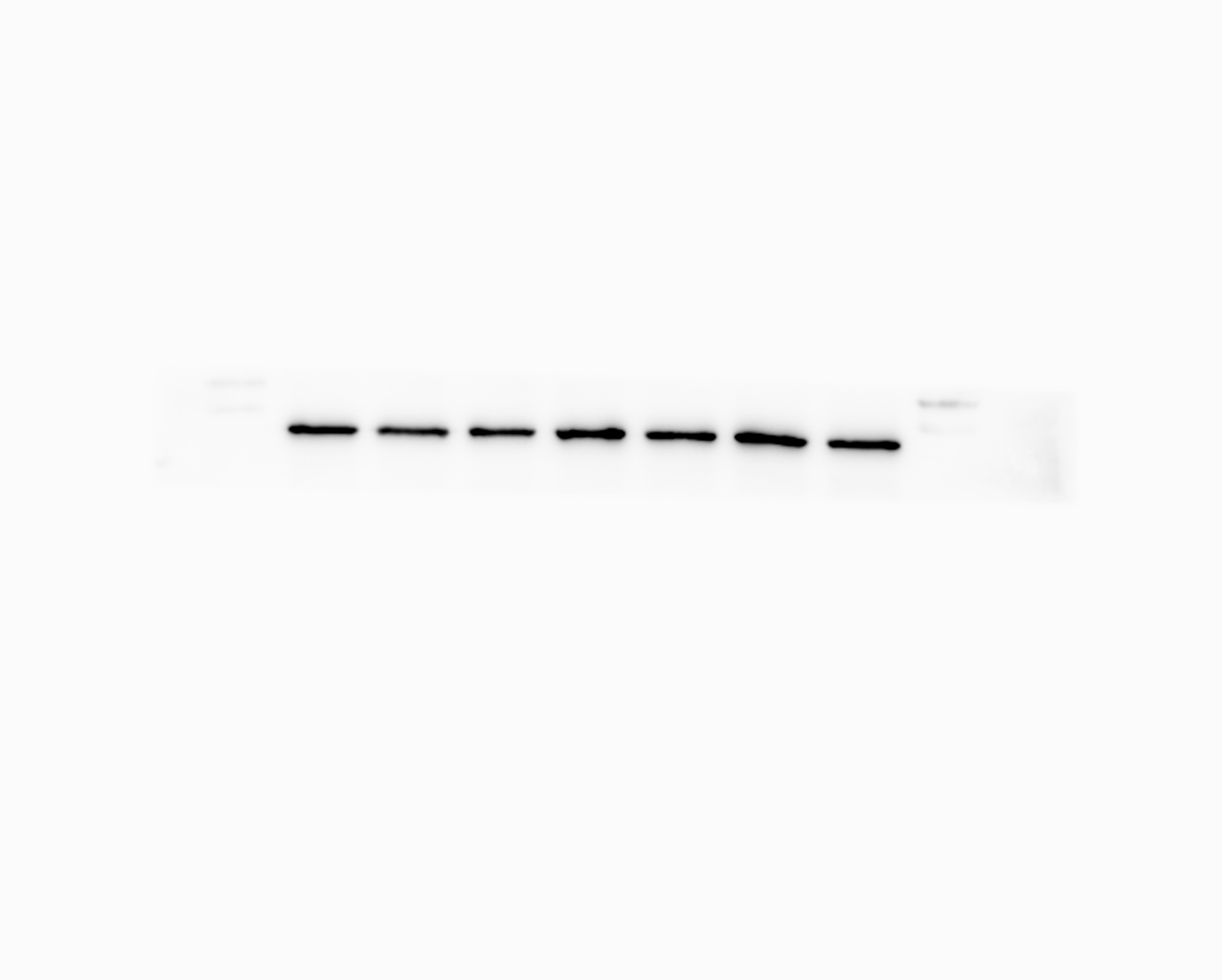

Supplement: Figure 3—figure supplement 2—source data 1. [file elife-97827-fig3-figsupp2-data1.zip › Figure 3-figure supplement 2-source data 3.2 .tiff]

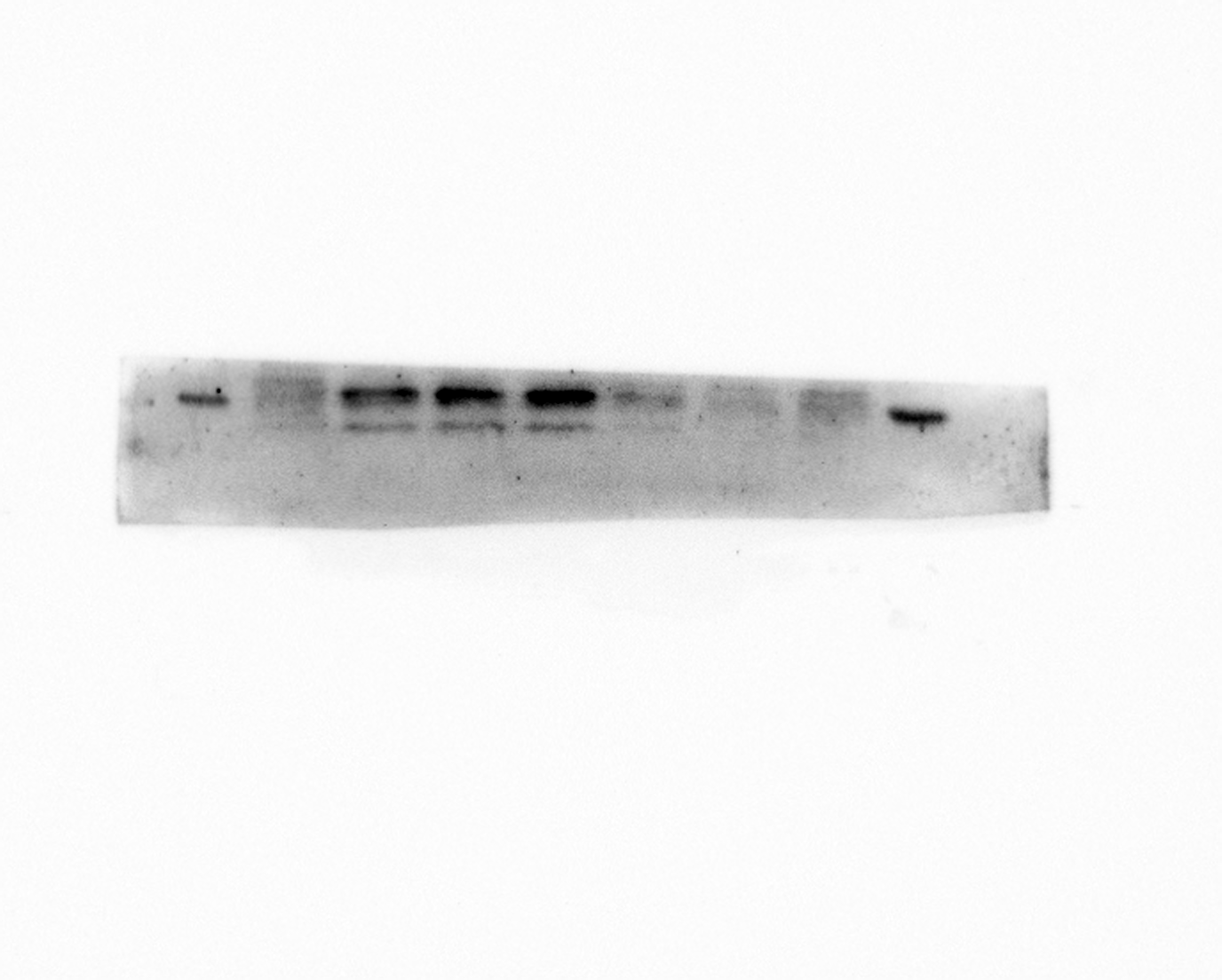

Supplement: Figure 3—figure supplement 2—source data 1. [file elife-97827-fig3-figsupp2-data1.zip › Figure 3-figure supplement 2-source data 3.3 .tiff]

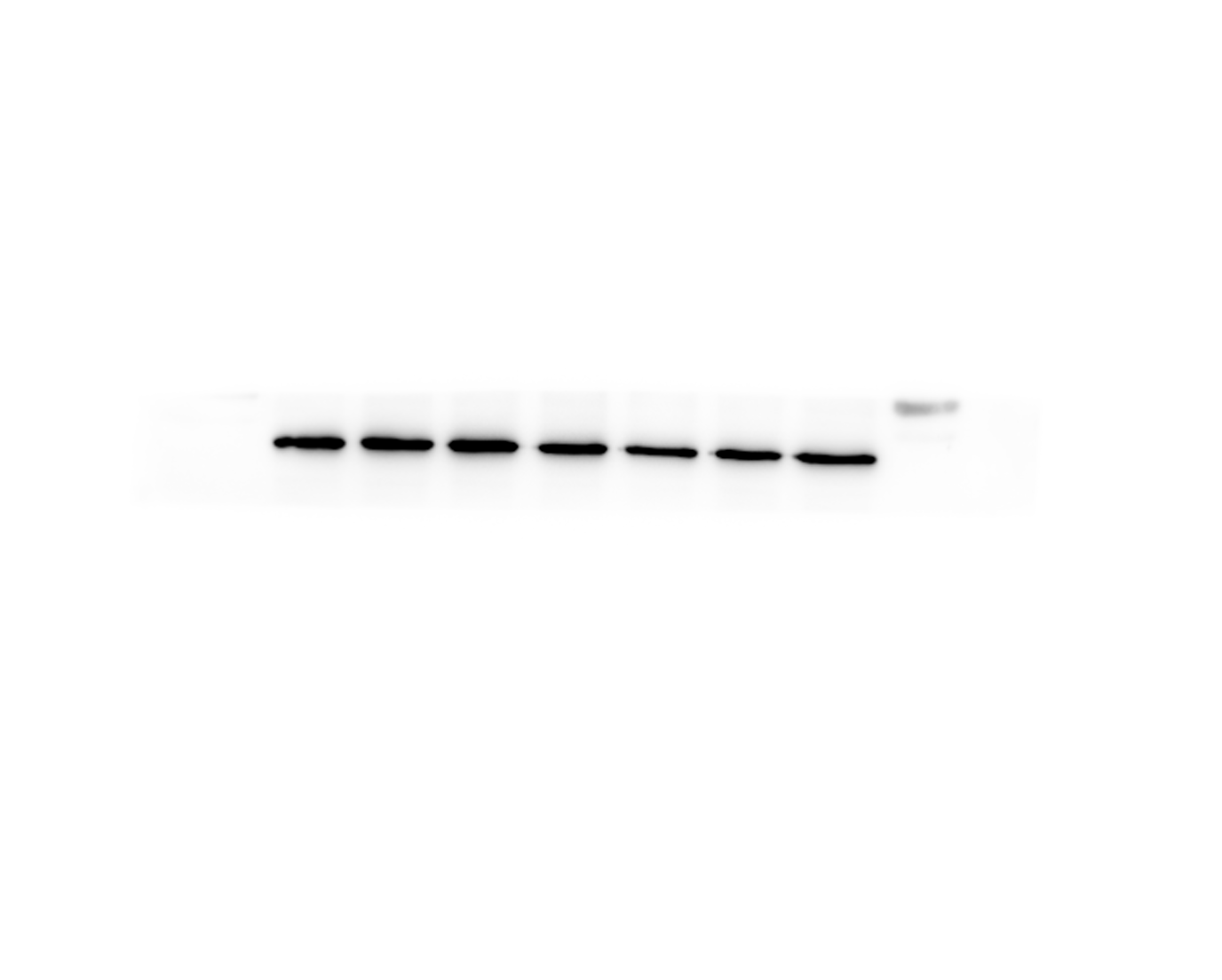

Supplement: Figure 3—figure supplement 2—source data 1. [file elife-97827-fig3-figsupp2-data1.zip › Figure 3-figure supplement 2-source data 3.4 .tiff]

Figure 3-figure supplement 2D

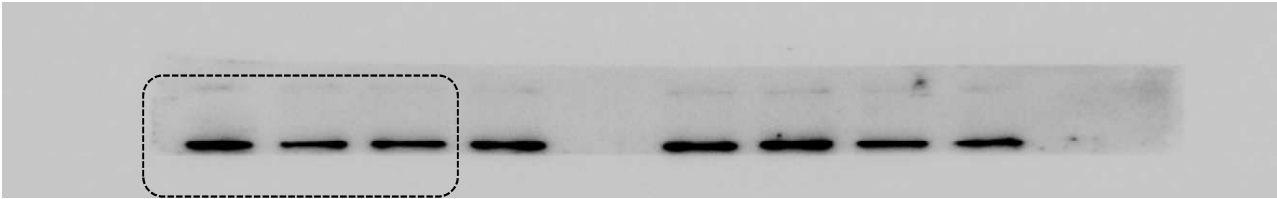

CPT1A

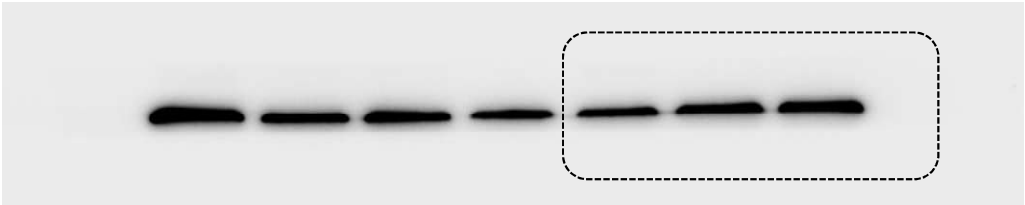

$\beta$ -actin

Supplement: Figure 3—figure supplement 2—source data 2. [file elife-97827-fig3-figsupp2-data2.zip › Figure 3-figure supplement 2-source data 1.pdf]

**Figure 3-figure supplement 2E**

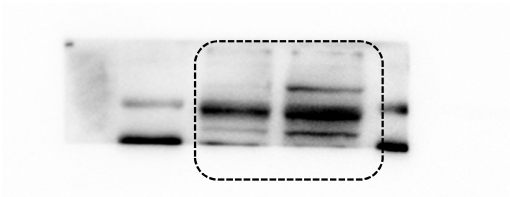

**CPT1A**

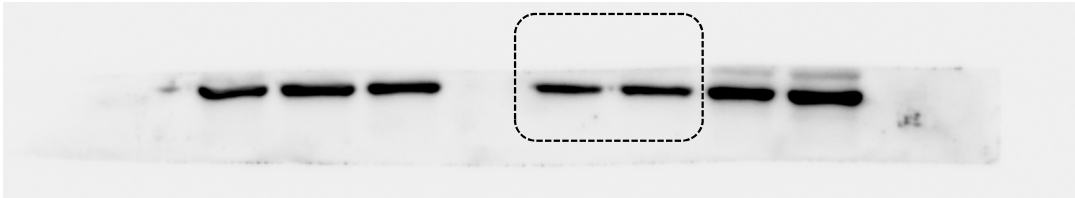

**β-actin**

Supplement: Figure 3—figure supplement 2—source data 2. [file elife-97827-fig3-figsupp2-data2.zip › Figure 3-figure supplement 2-source data 2.pdf]

**Figure 3-figure supplement 2F**

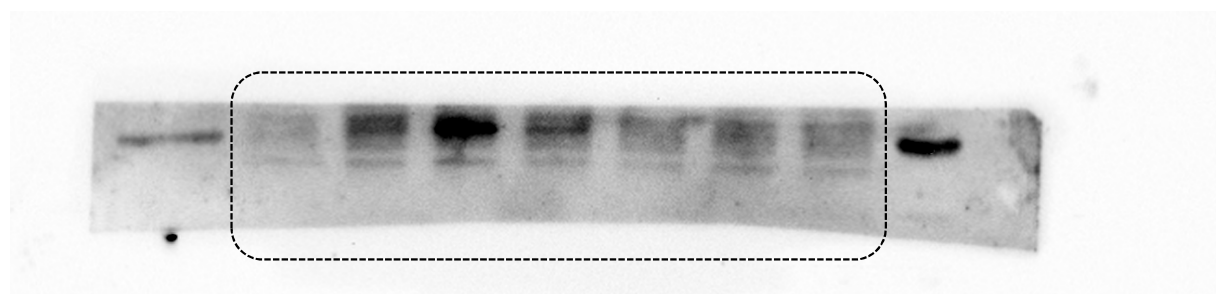

**γ-H2A.X**

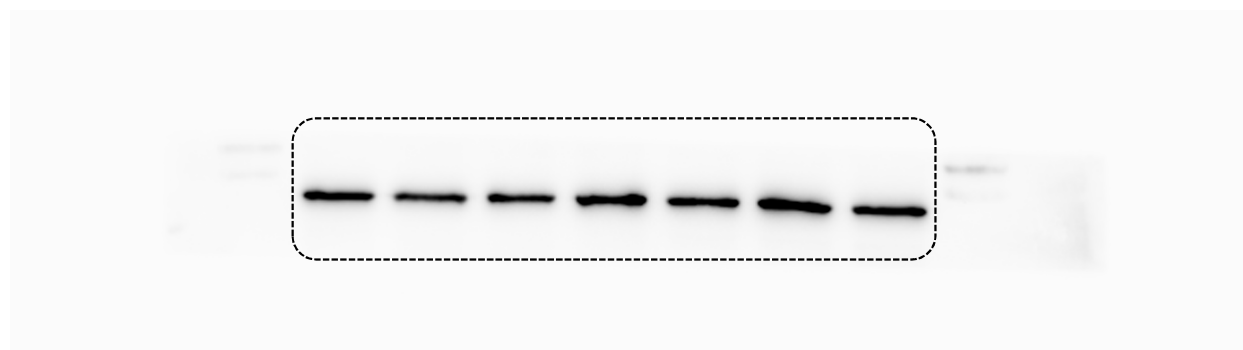

**β-actin**

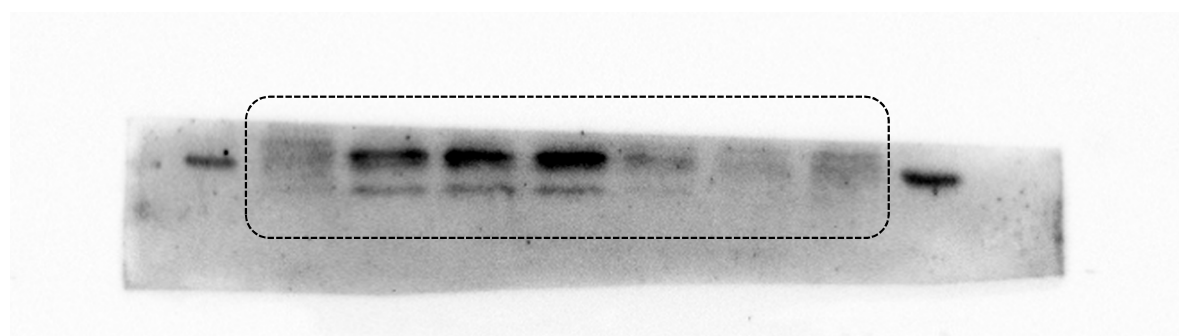

**γ-H2A.X**

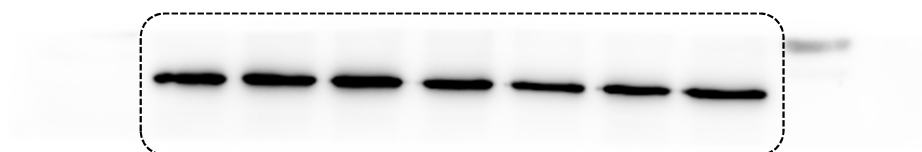

**β-actin**

Supplement: Figure 3—figure supplement 2—source data 2. [file elife-97827-fig3-figsupp2-data2.zip › Figure 3-figure supplement 2-source data 3.pdf]

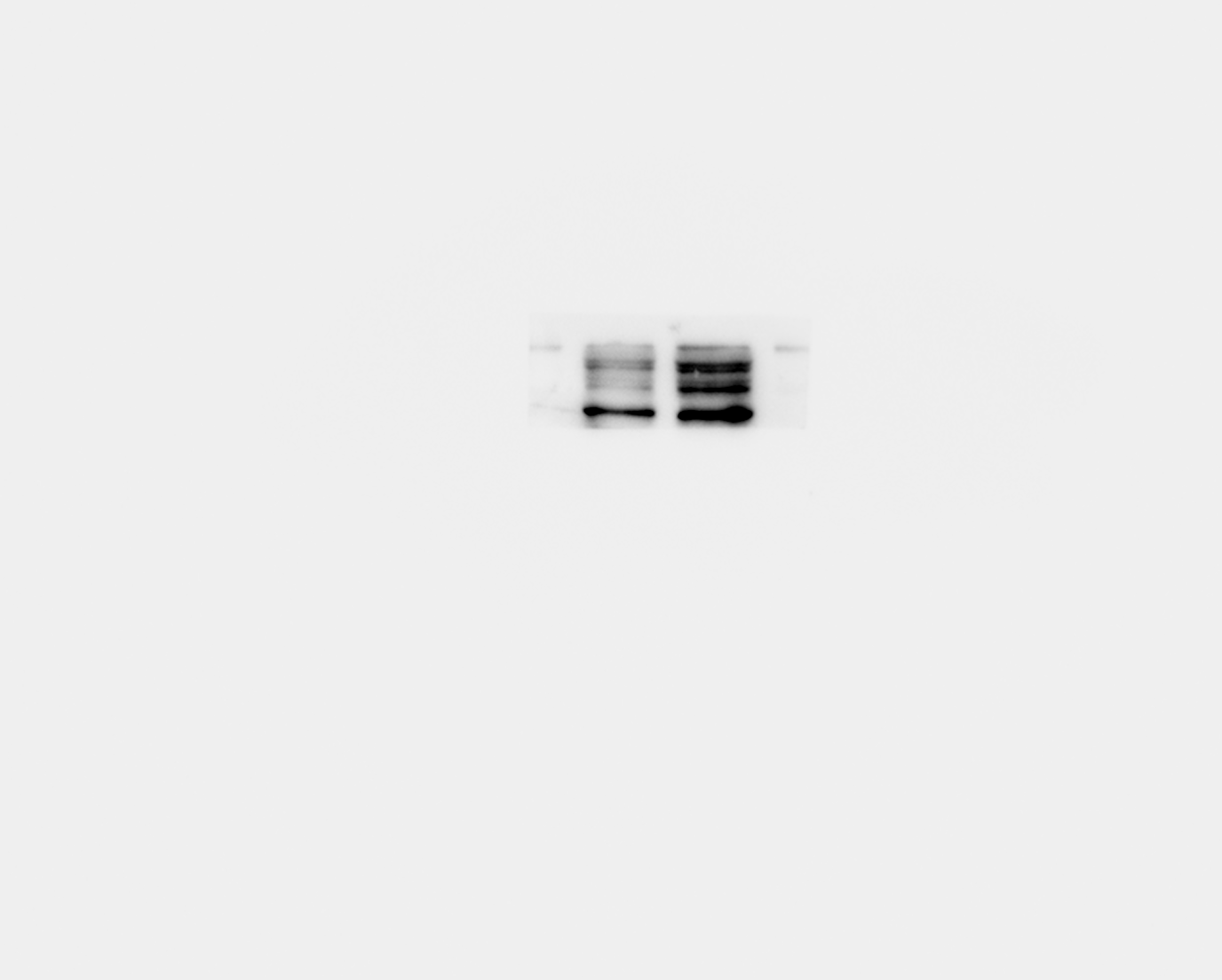

Supplement: Figure 6—source data 1. [file elife-97827-fig6-data1.zip › Figure 6-source data 1.1 .tiff]

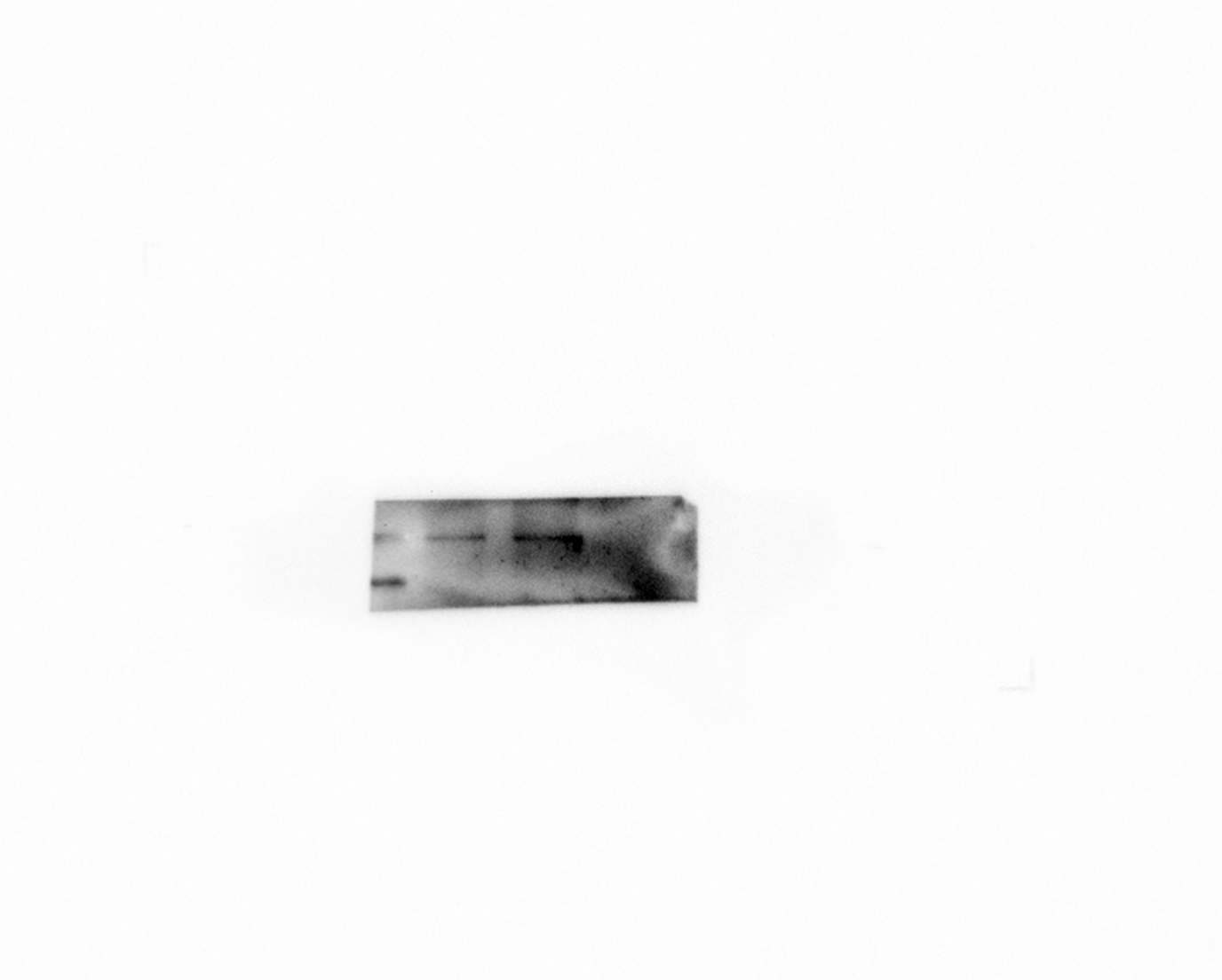

Supplement: Figure 6—source data 1. [file elife-97827-fig6-data1.zip › Figure 6-source data 1.10 .tiff]

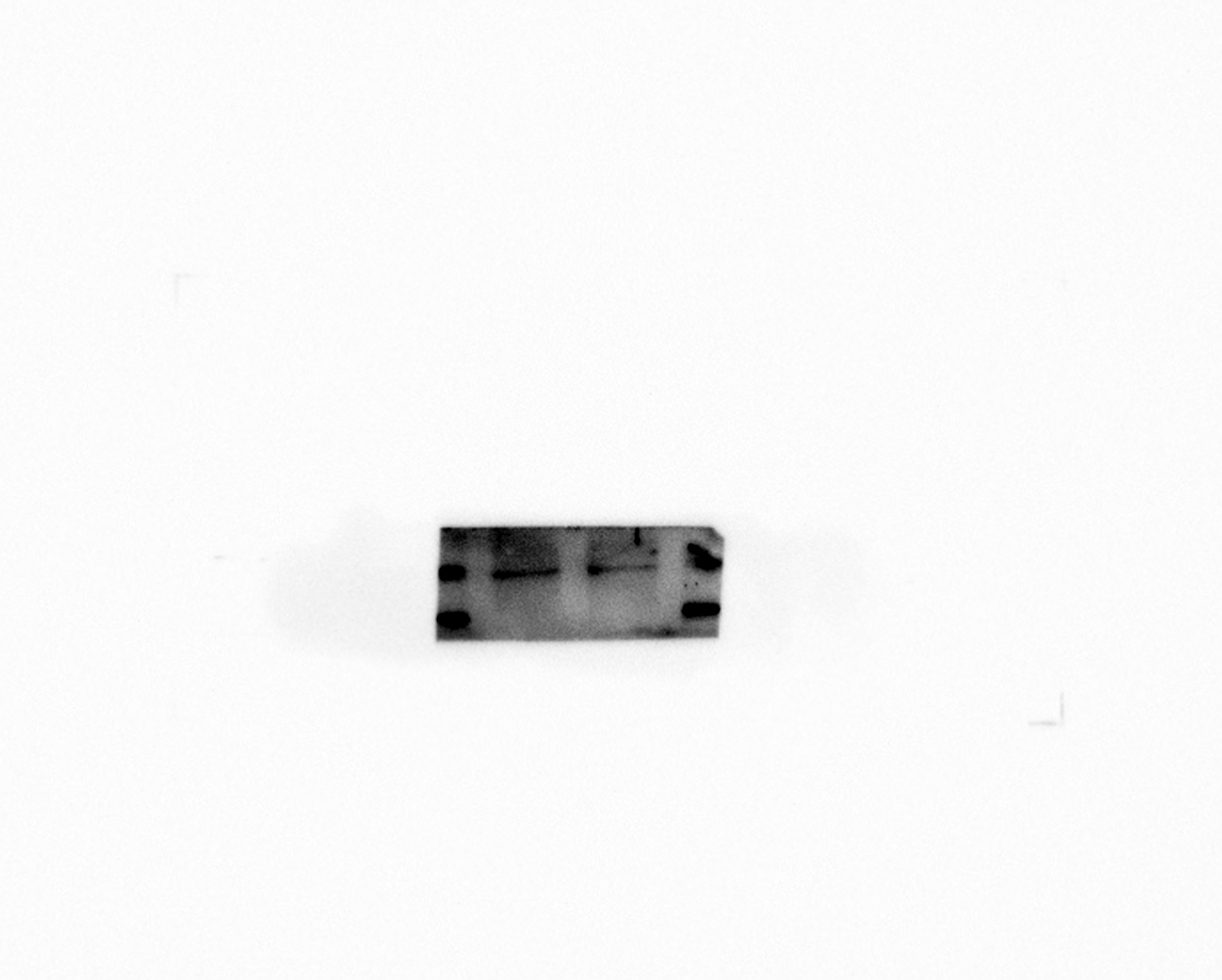

Supplement: Figure 6—source data 1. [file elife-97827-fig6-data1.zip › Figure 6-source data 1.11 .tiff]

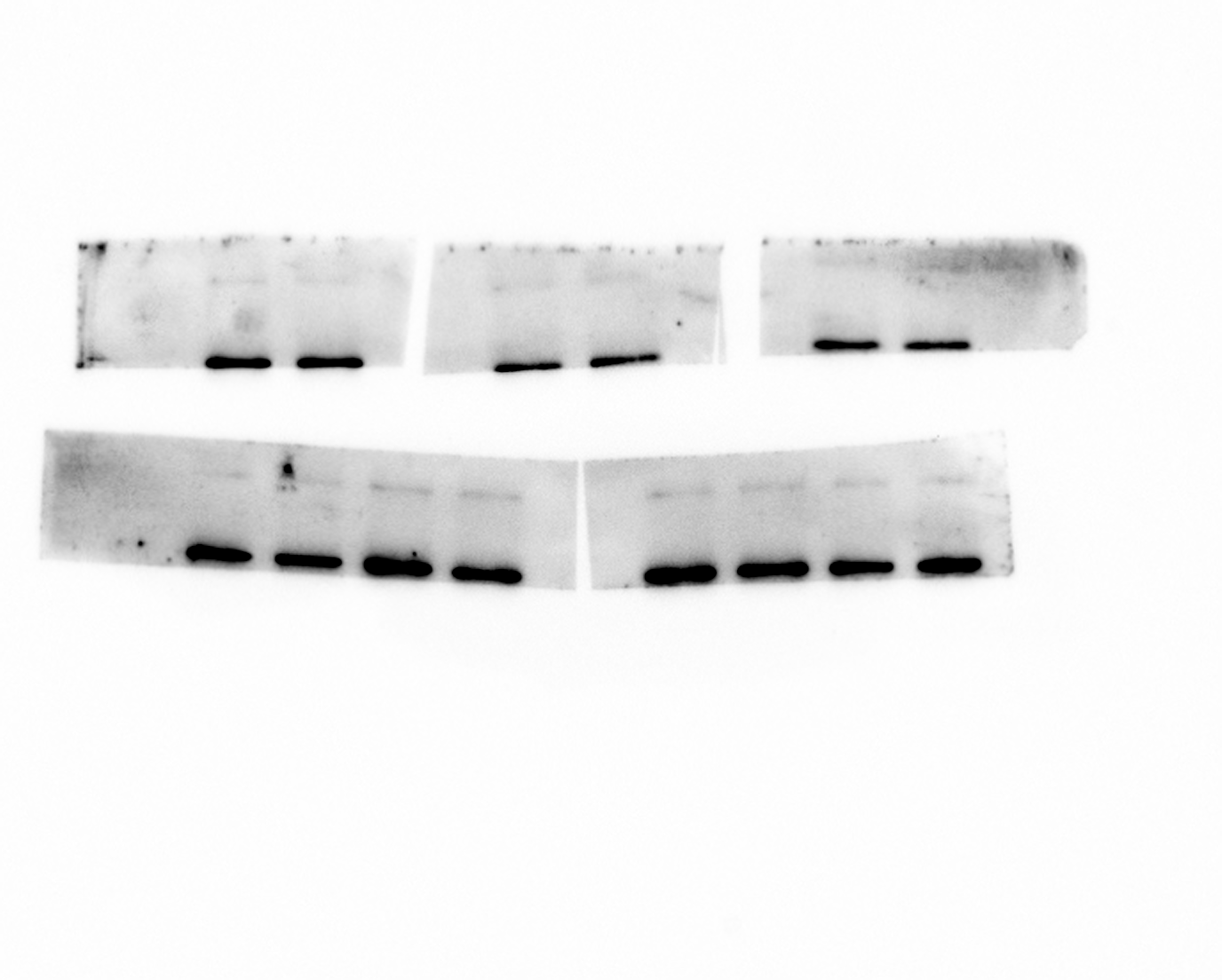

Supplement: Figure 6—source data 1. [file elife-97827-fig6-data1.zip › Figure 6-source data 1.12 .tiff]

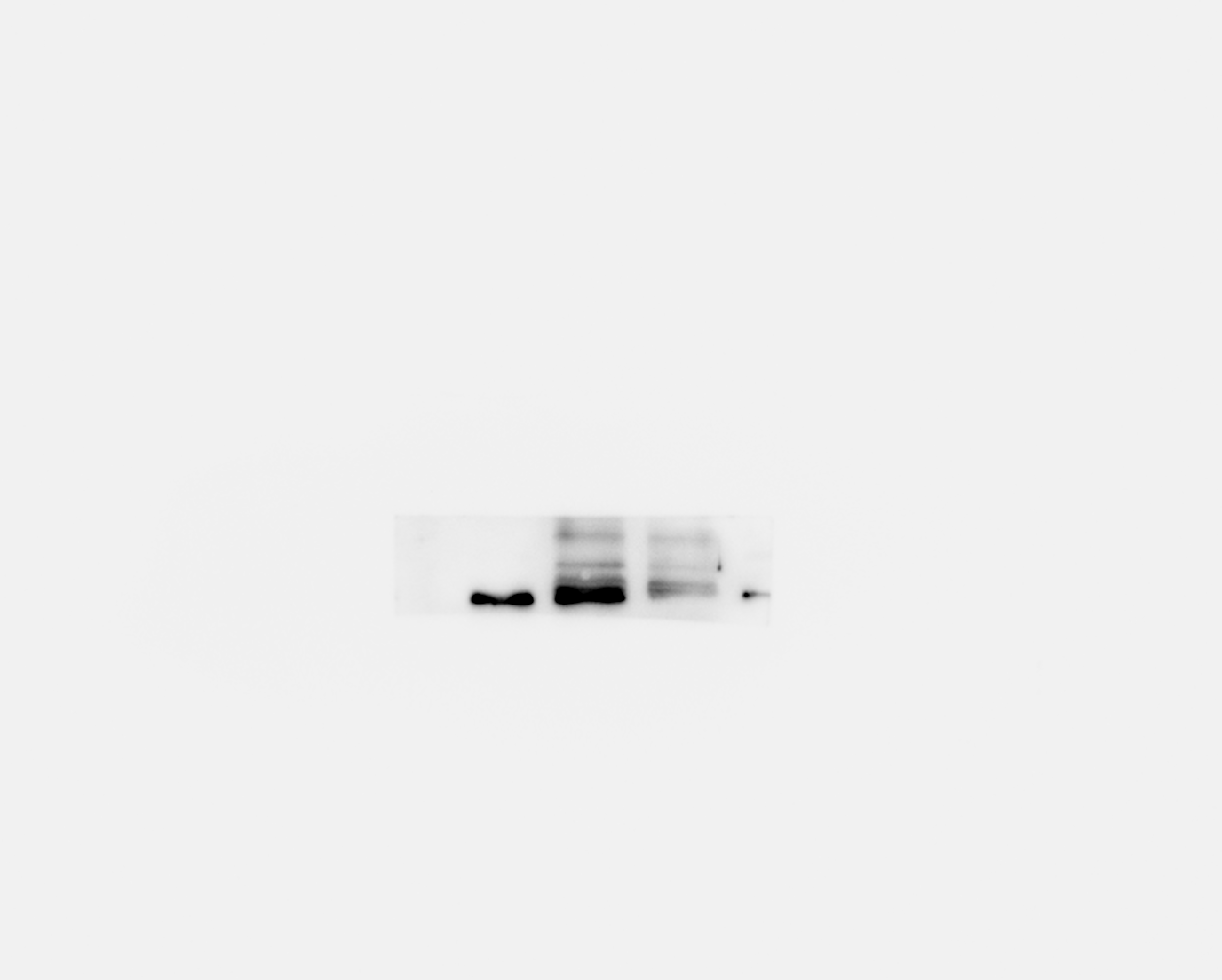

Supplement: Figure 6—source data 1. [file elife-97827-fig6-data1.zip › Figure 6-source data 1.2 .tiff]

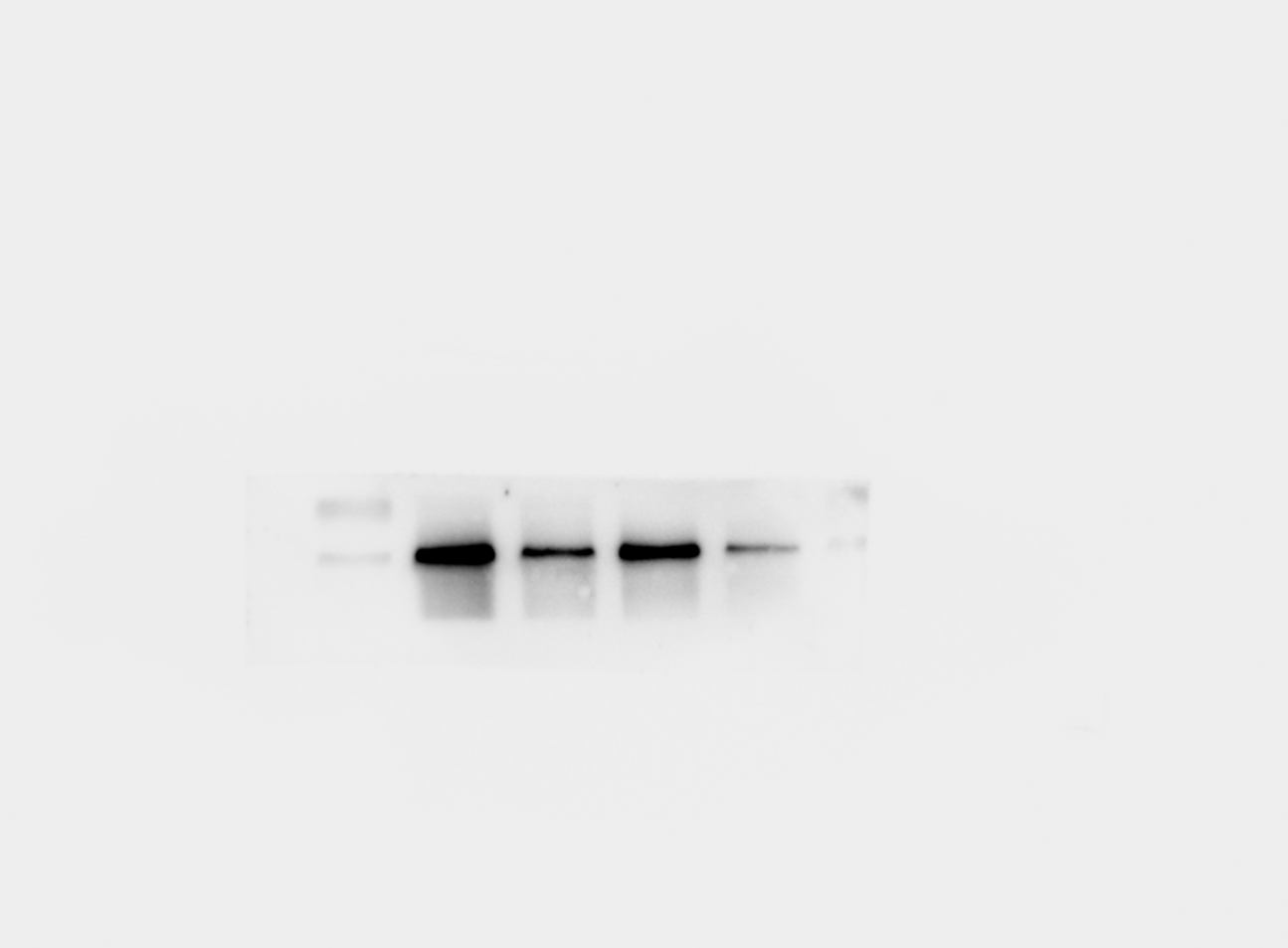

Supplement: Figure 6—source data 1. [file elife-97827-fig6-data1.zip › Figure 6-source data 1.3 .tiff]

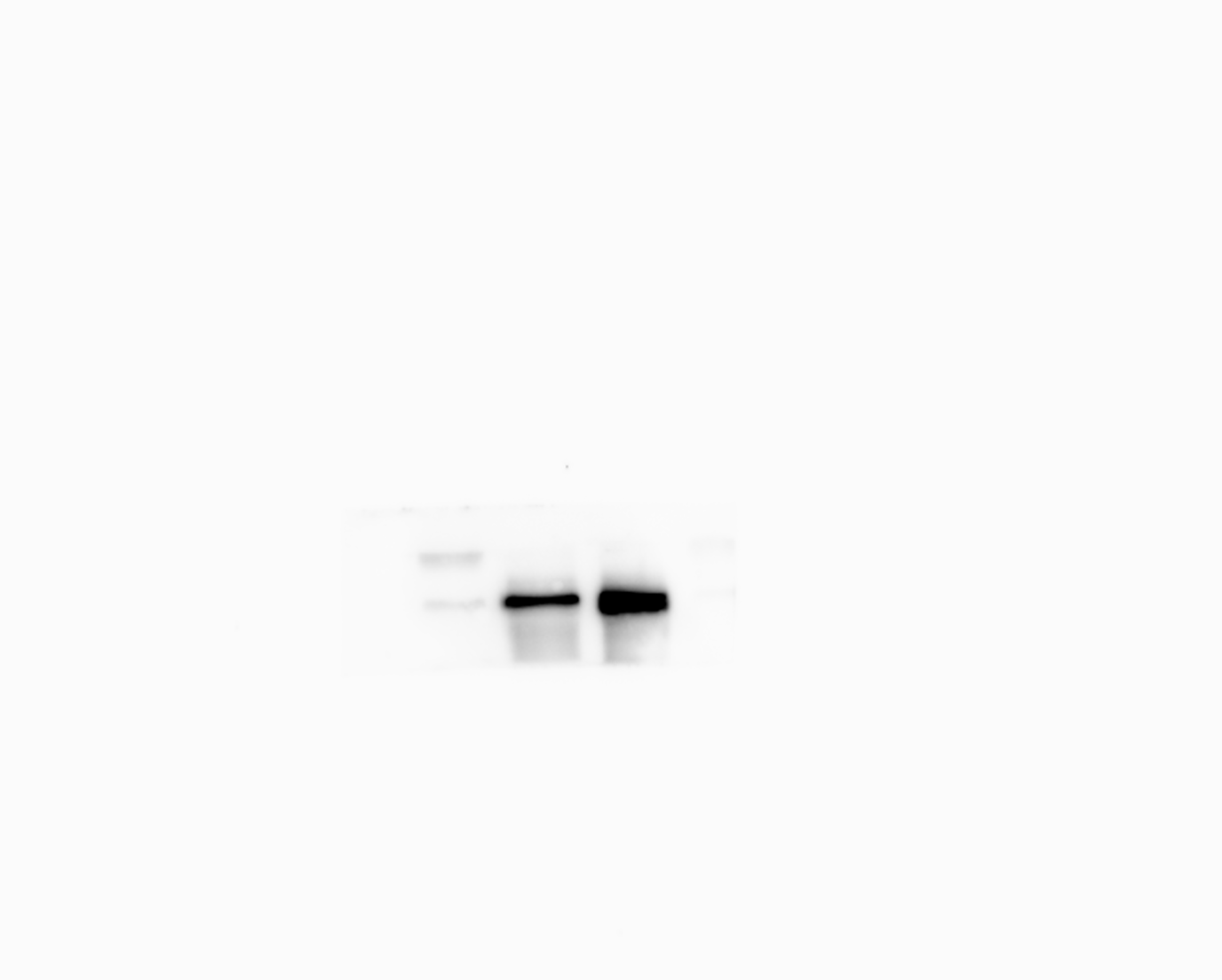

Supplement: Figure 6—source data 1. [file elife-97827-fig6-data1.zip › Figure 6-source data 1.4 .tiff]

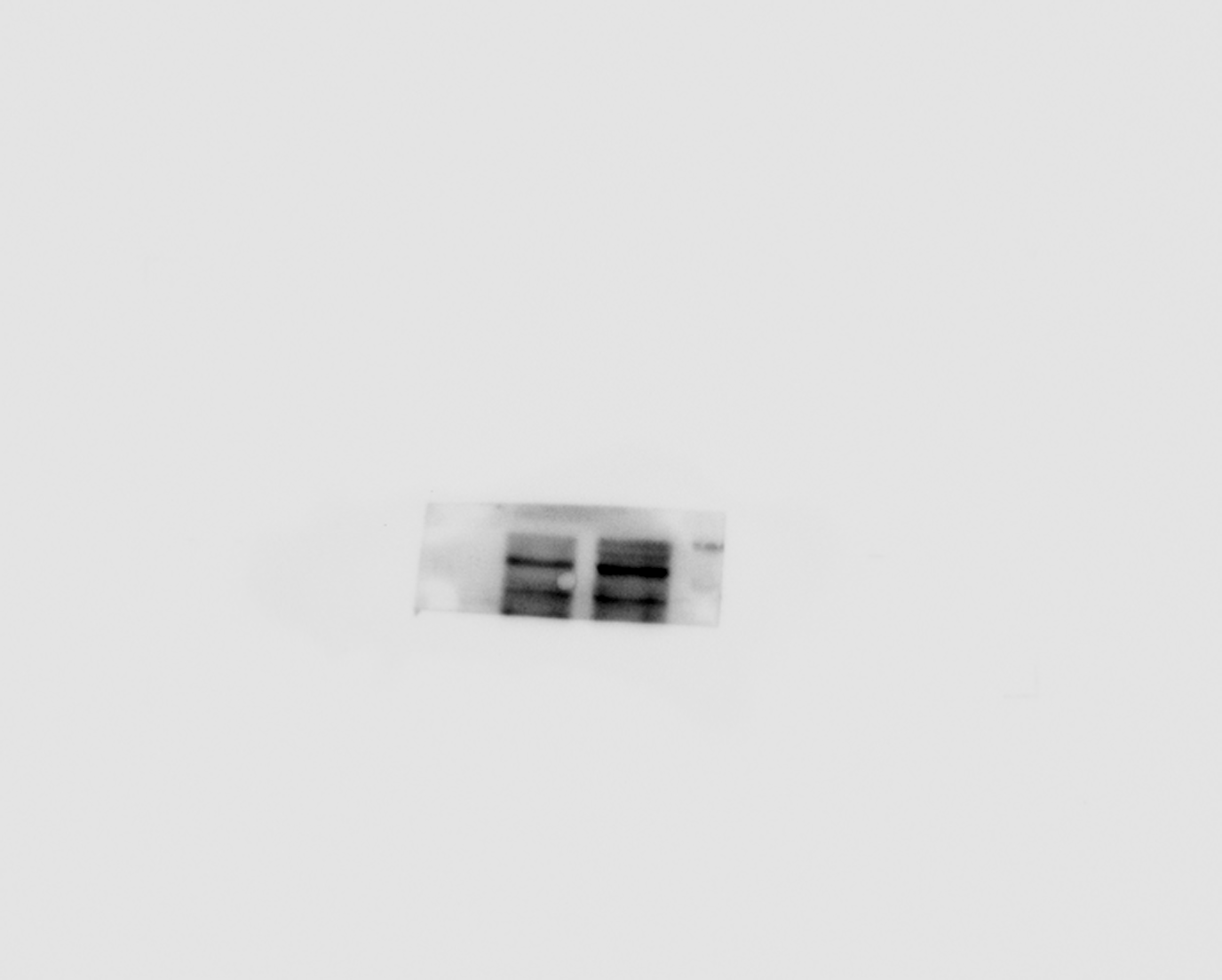

Supplement: Figure 6—source data 1. [file elife-97827-fig6-data1.zip › Figure 6-source data 1.5 .tiff]

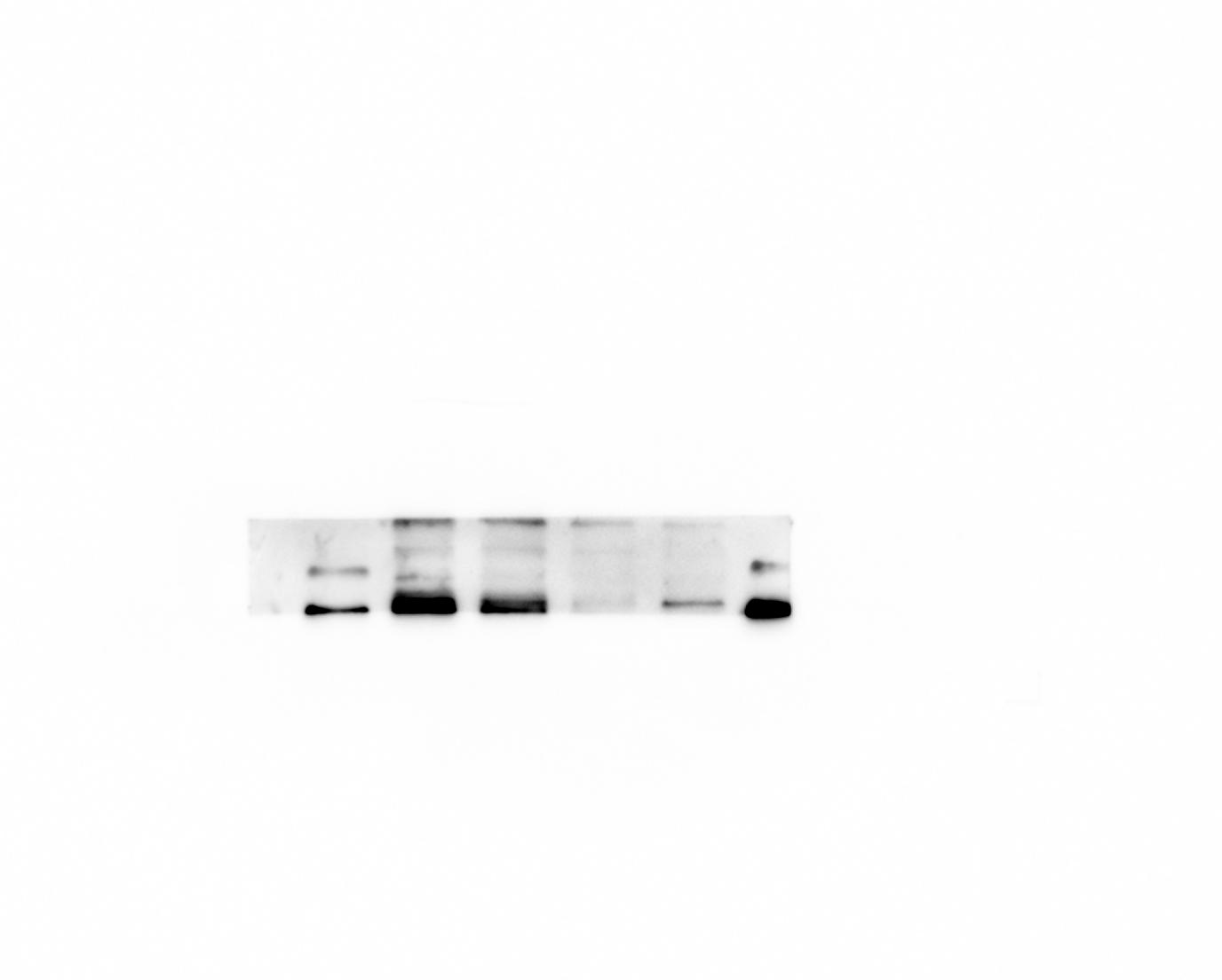

Supplement: Figure 6—source data 1. [file elife-97827-fig6-data1.zip › Figure 6-source data 1.6 .tiff]

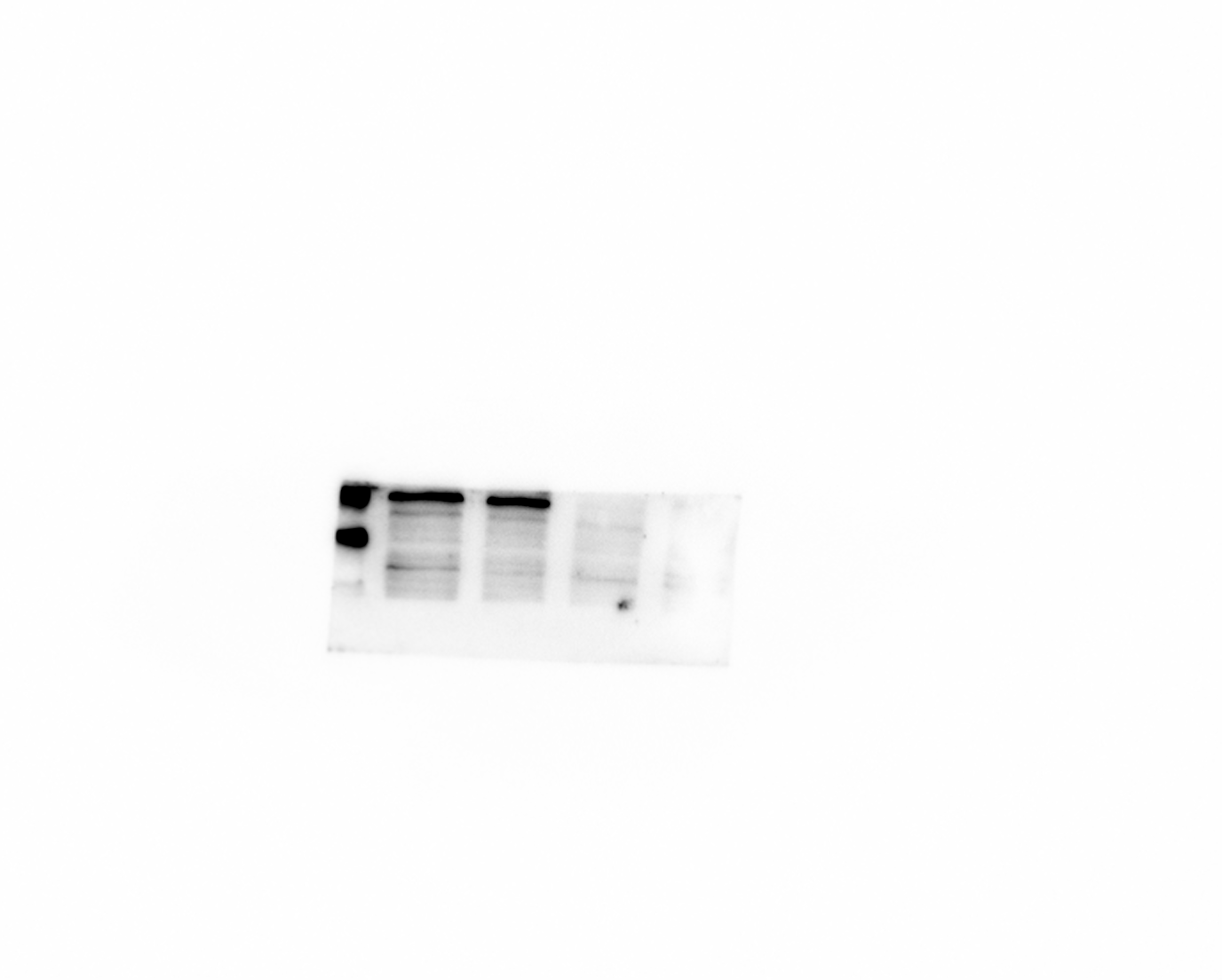

Supplement: Figure 6—source data 1. [file elife-97827-fig6-data1.zip › Figure 6-source data 1.7 .tiff]

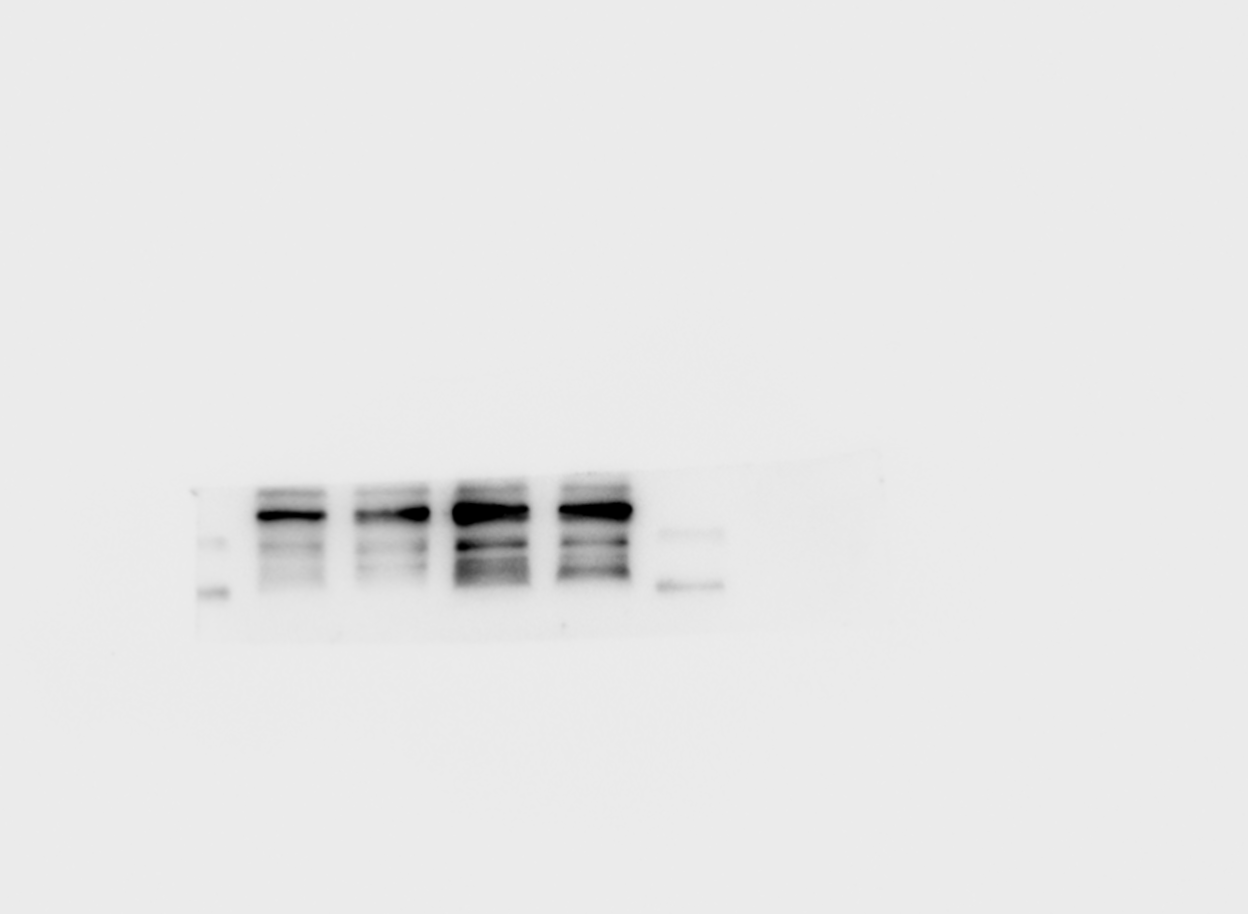

Supplement: Figure 6—source data 1. [file elife-97827-fig6-data1.zip › Figure 6-source data 1.8 .tiff]

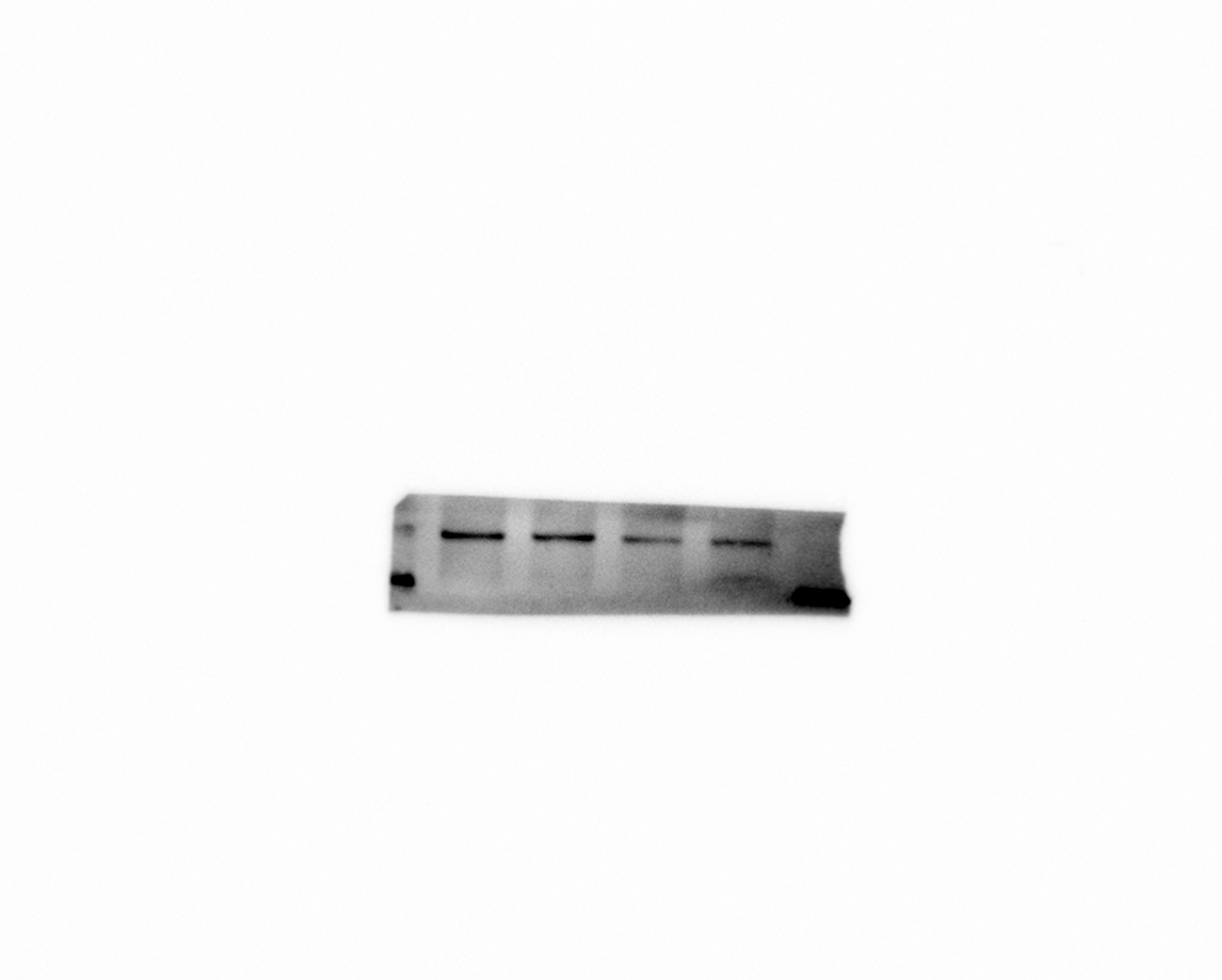

Supplement: Figure 6—source data 1. [file elife-97827-fig6-data1.zip › Figure 6-source data 1.9 .tiff]

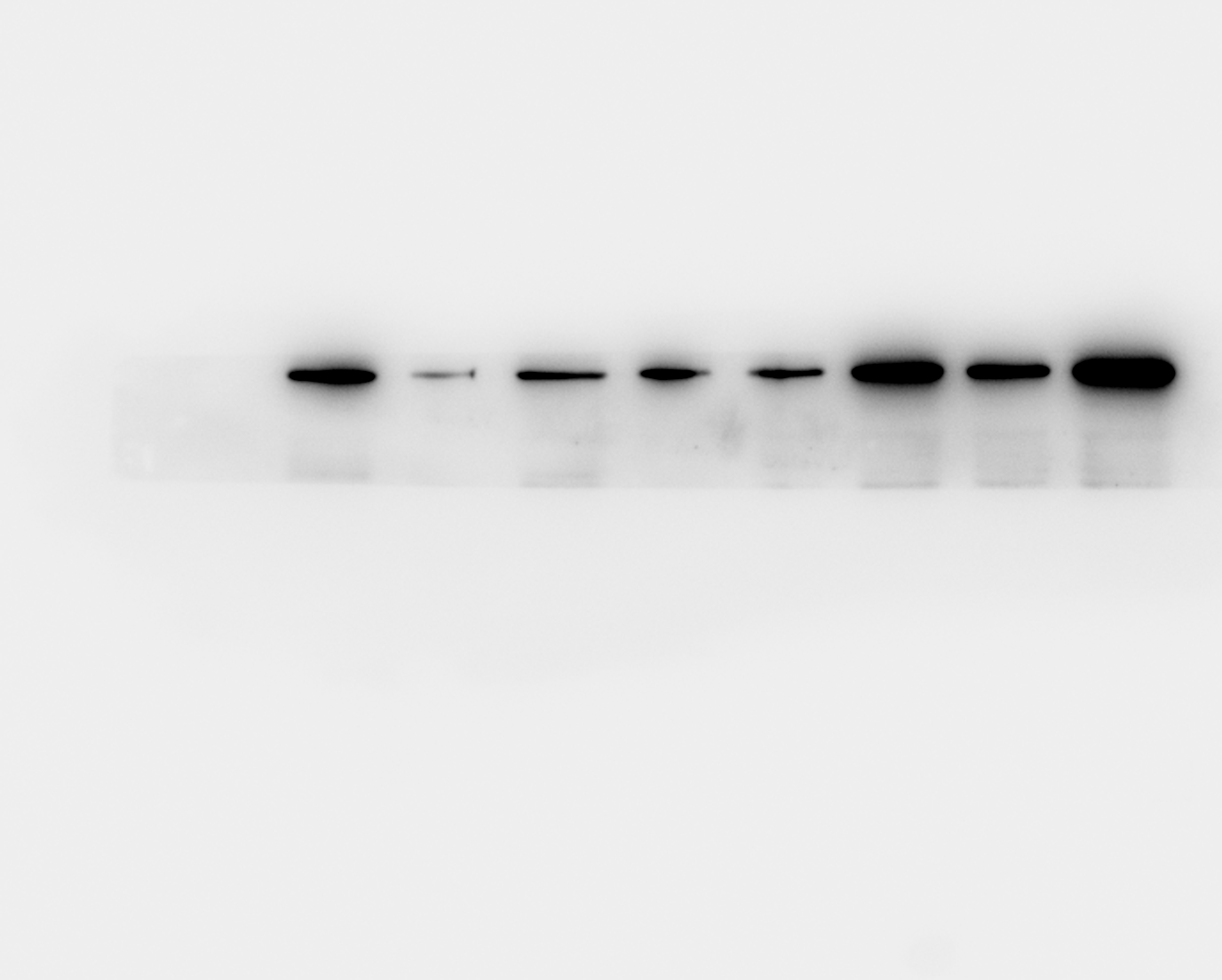

Supplement: Figure 6—source data 1. [file elife-97827-fig6-data1.zip › Figure 6-source data 2.1 .tiff]

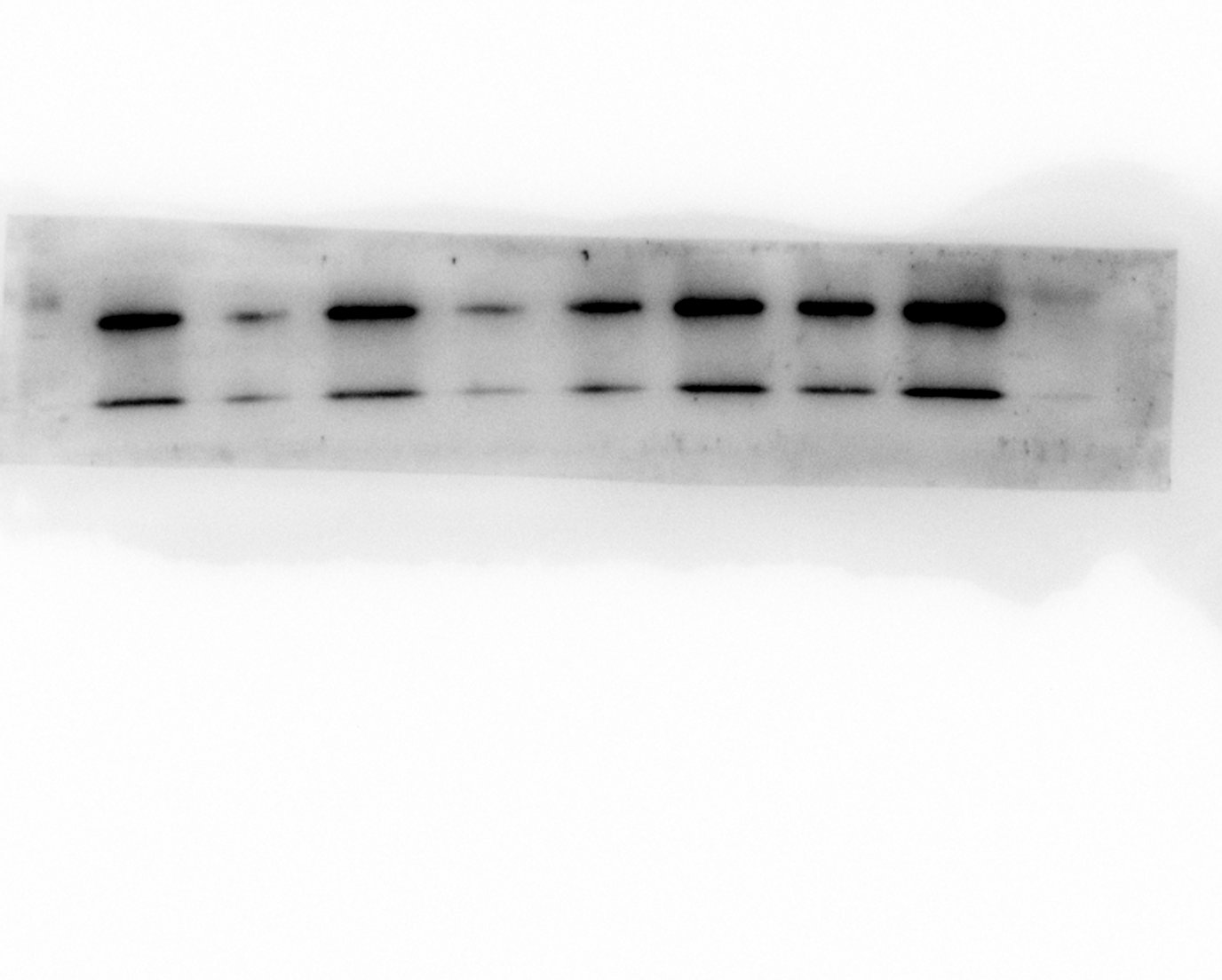

Supplement: Figure 6—source data 1. [file elife-97827-fig6-data1.zip › Figure 6-source data 2.2 .tiff]

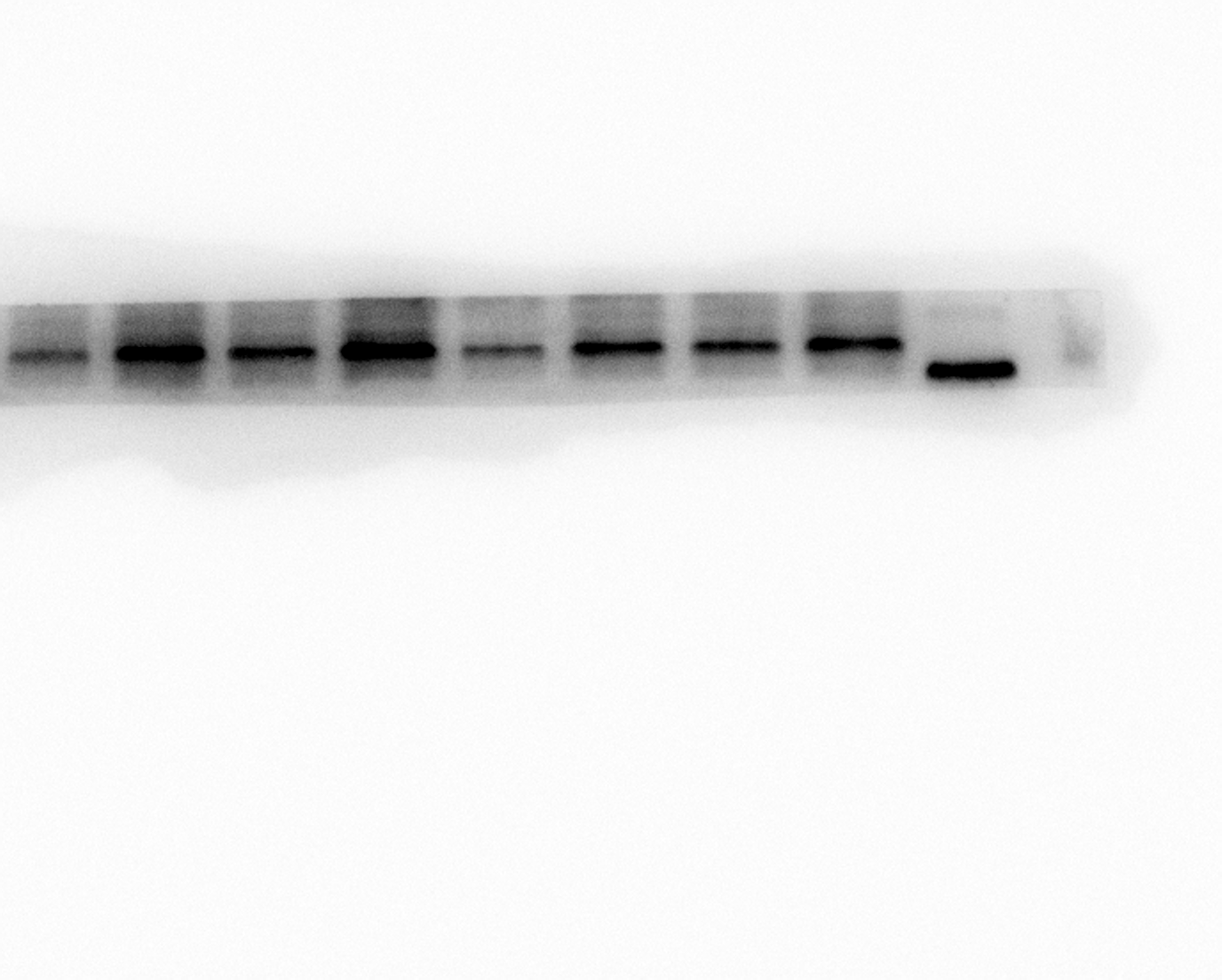

Supplement: Figure 6—source data 1. [file elife-97827-fig6-data1.zip › Figure 6-source data 2.3 .tiff]

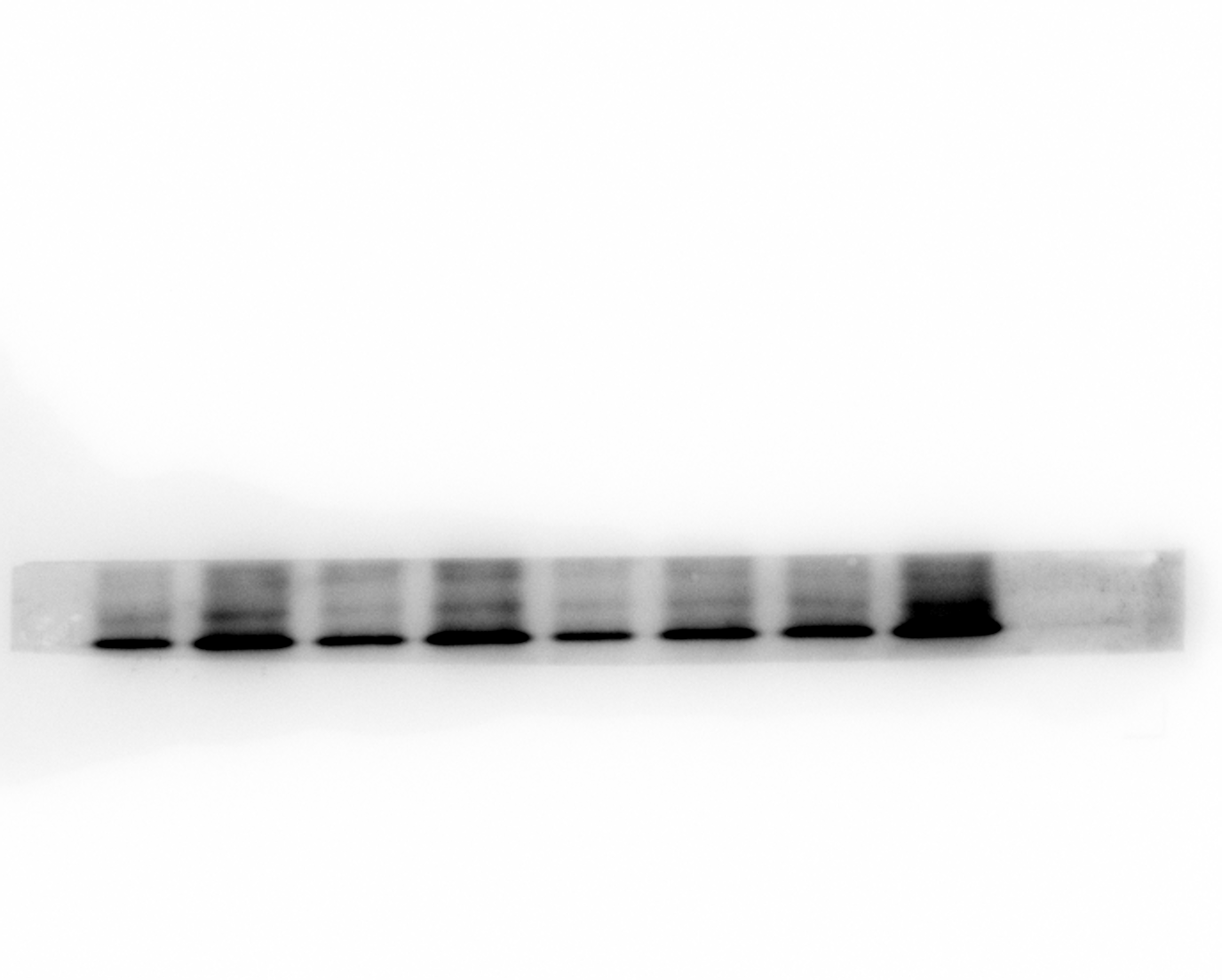

Supplement: Figure 6—source data 1. [file elife-97827-fig6-data1.zip › Figure 6-source data 2.4 .tiff]

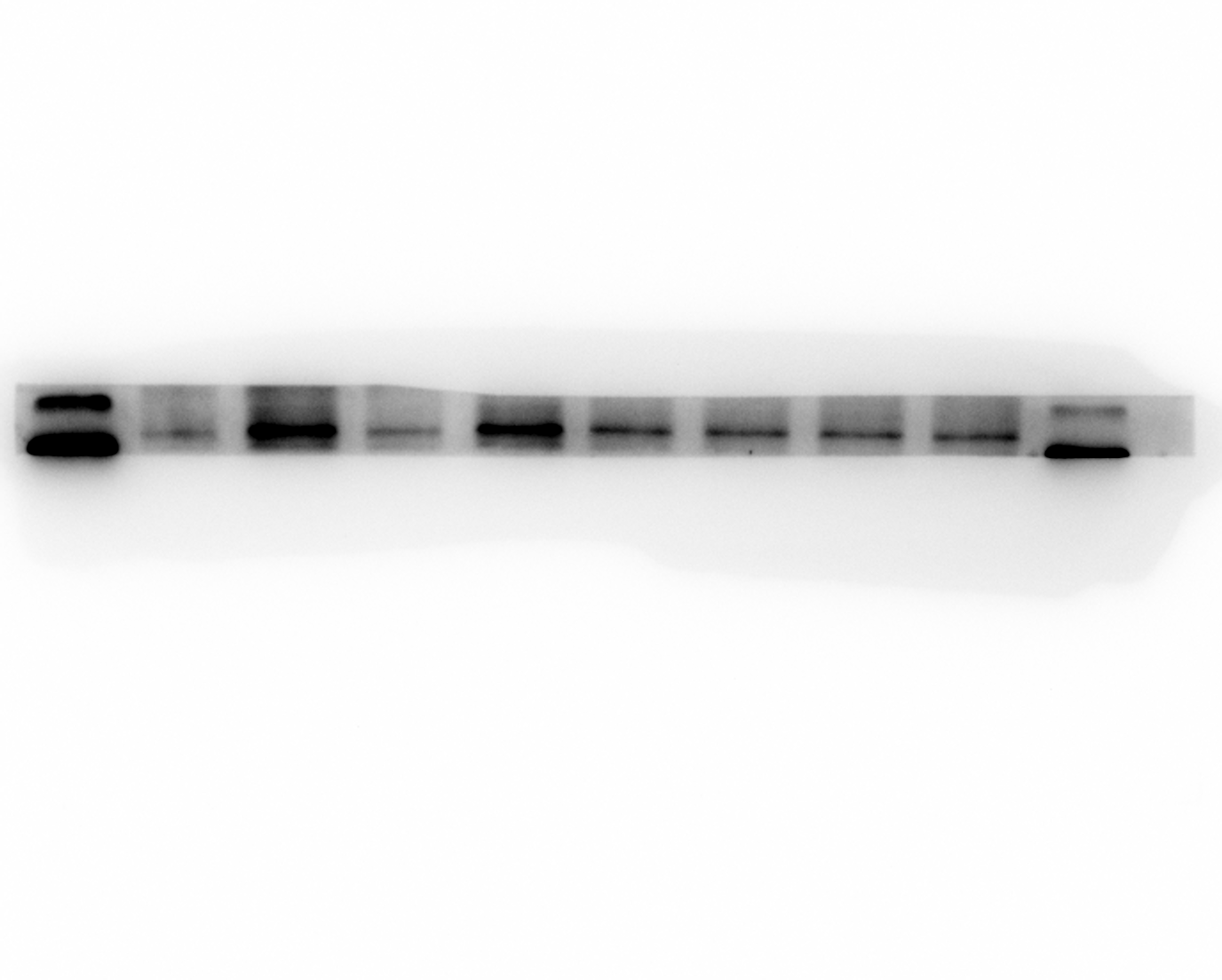

Supplement: Figure 6—source data 1. [file elife-97827-fig6-data1.zip › Figure 6-source data 2.5 .tiff]

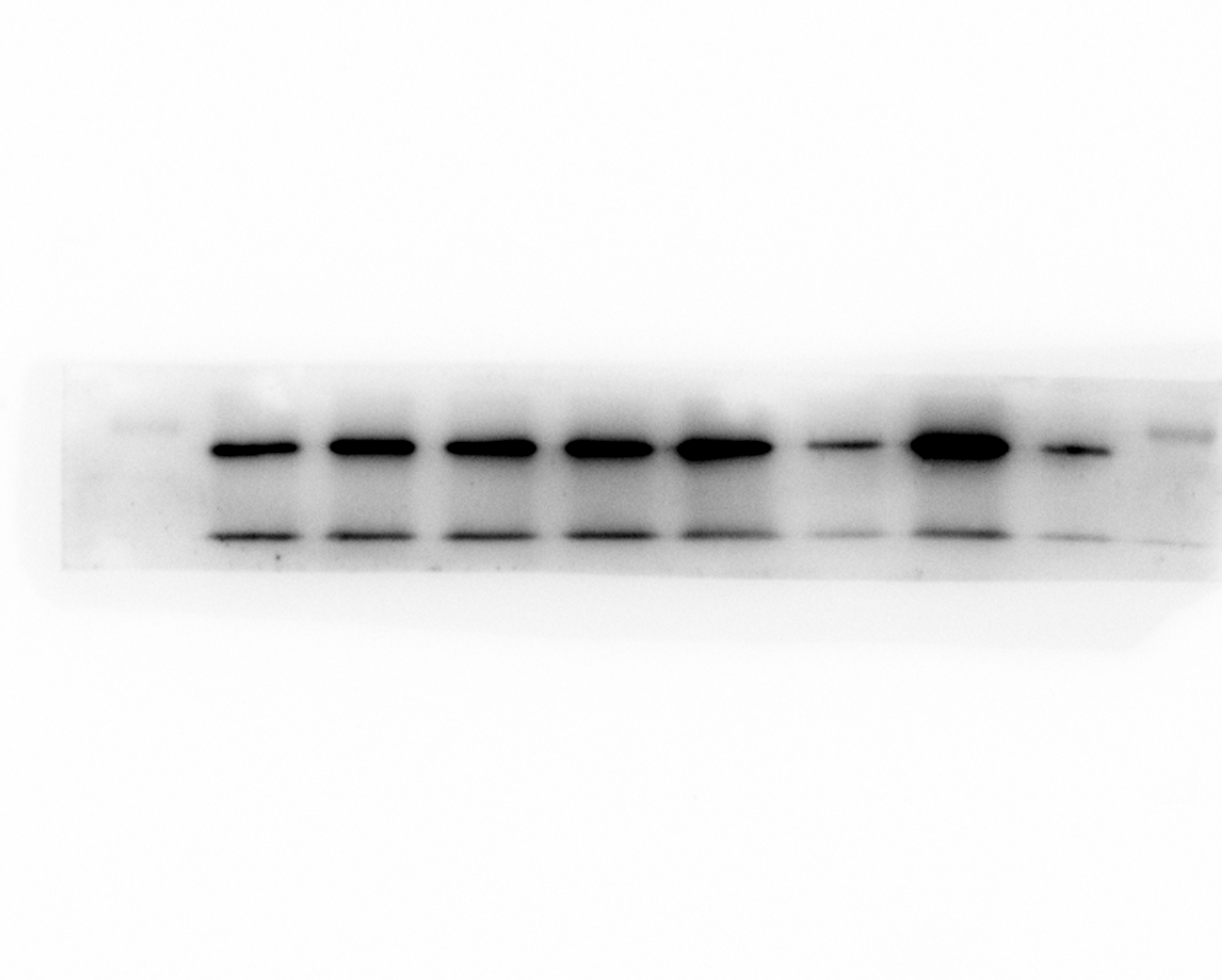

Supplement: Figure 6—source data 1. [file elife-97827-fig6-data1.zip › Figure 6-source data 2.6 .tiff]

**Figure 6A**

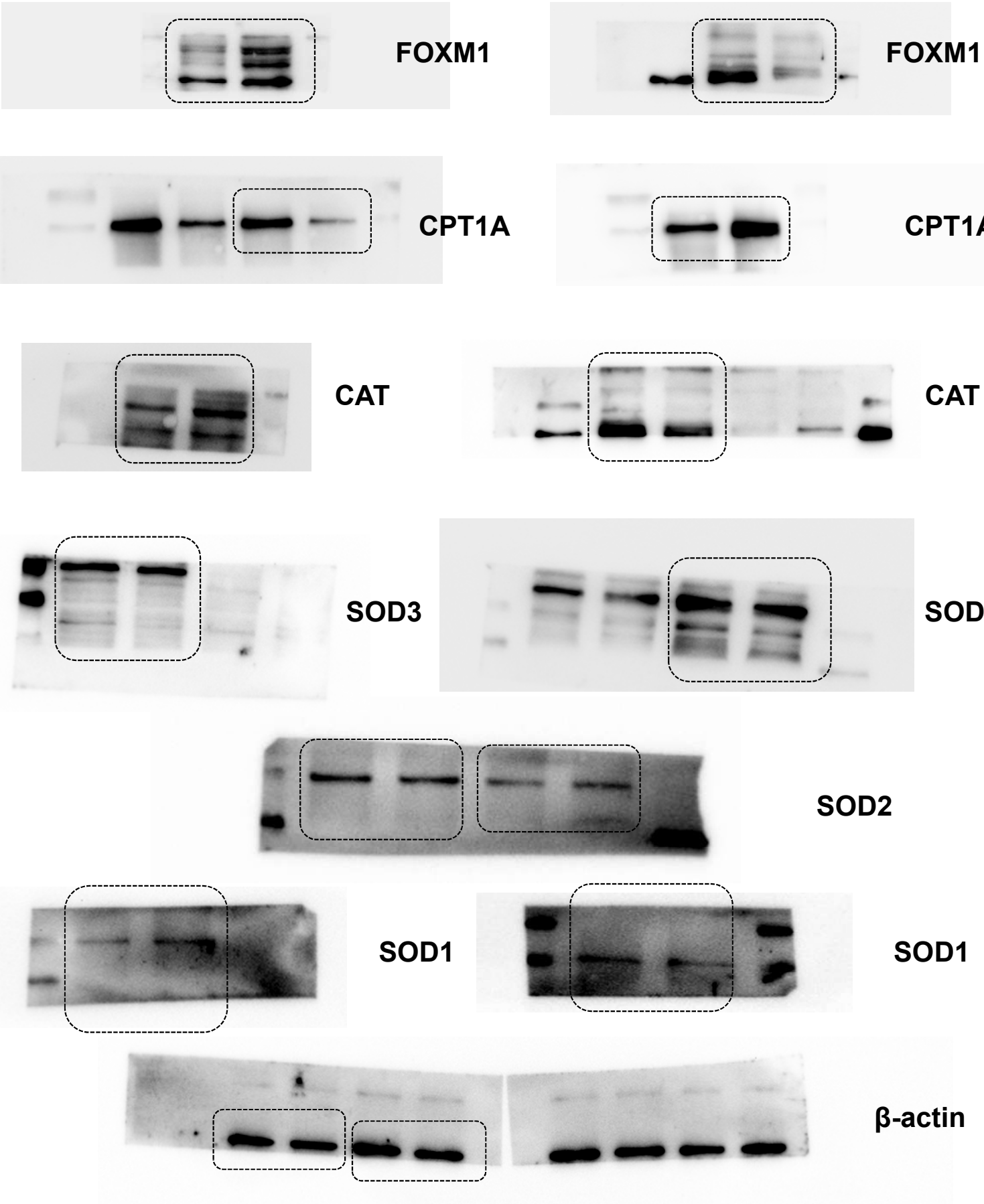

Supplement: Figure 6—source data 2. [file elife-97827-fig6-data2.zip › Figure 6-source data 1.pdf]

**Figure 6D**

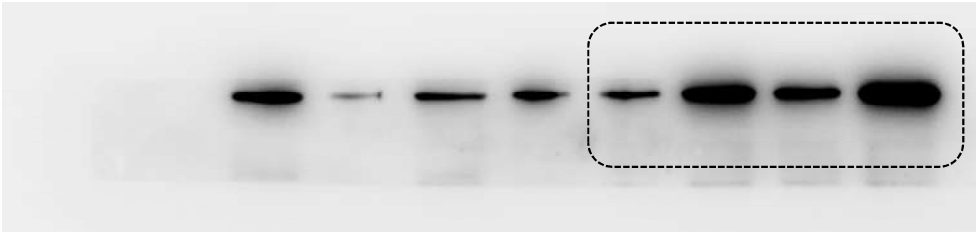

**FOXM1**

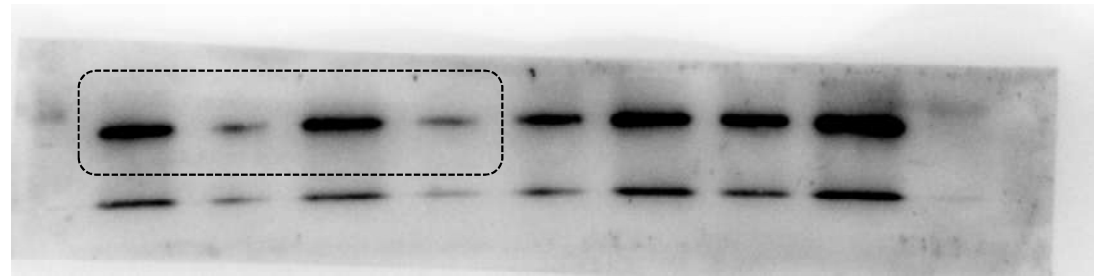

**CPT1A**

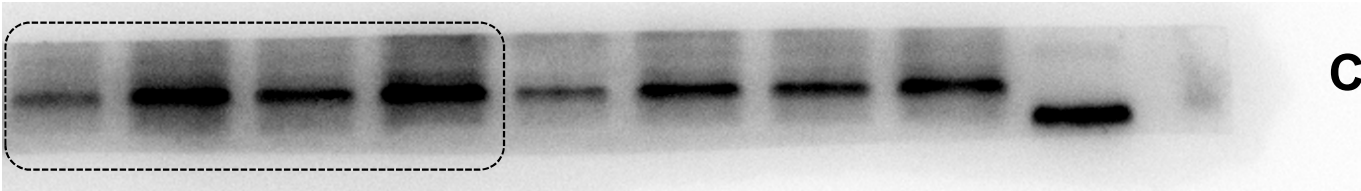

**CAT**

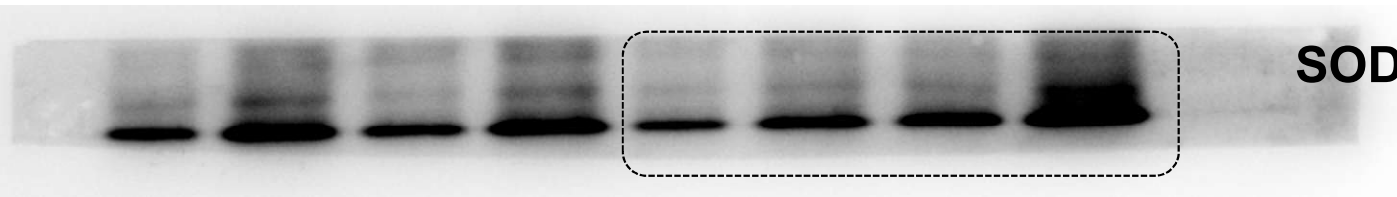

**SOD2**

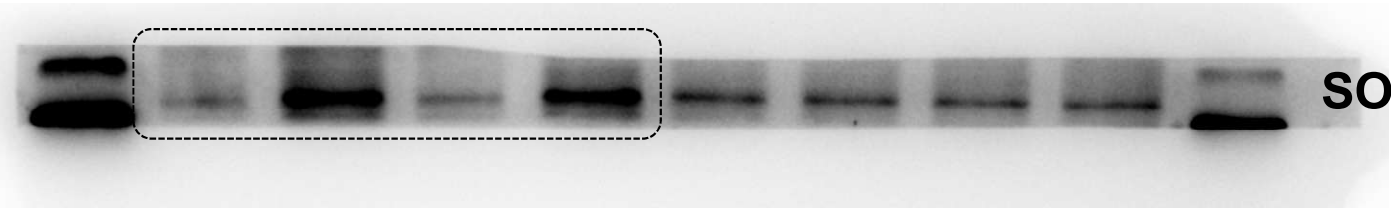

**SOD1**

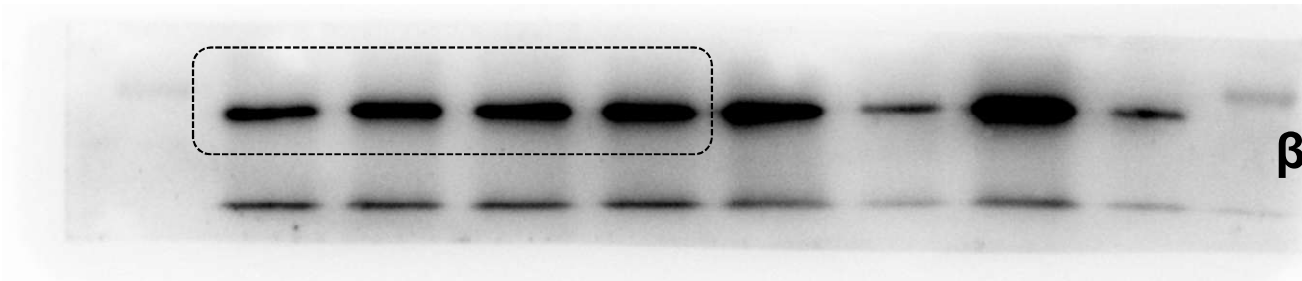

**$\beta$ -actin**

Supplement: Figure 6—source data 2. [file elife-97827-fig6-data2.zip › Figure 6-source data 2.pdf]
