# Supplementary material for: CPT1A mediates radiation sensitivity in colorectal cancer
Source: eLife. 2024 Nov 28;13:RP97827. doi: 10.7554/eLife.97827 (PMC11604221; doi:10.7554/eLife.97827)
Supplement: Figure 1—source data 2. [file elife-97827-fig1-data2.zip › Figure 1- source data legends.docx]

**Figure 1- source data 1:** Western blots labelled with relevant bands analyzed in Figure 1C(anti-CPT1A and anti-β-actin)

**Figure 1- source data 1.1:** Original file for the western blot in Figure 1C(anti-CPT1A)

**Figure 1- source data 1.2:** Original file for the western blot in Figure 1C(anti-β-actin)

**Figure 1- source data 1.3:** Original file for the western blot in Figure 1C(anti-CPT1A)

**Figure 1- source data 1.4:** Original file for the western blot in Figure 1C(anti-β-actin)

**Figure 1- source data 1.5:** Original file for the western blot in Figure 1C(anti-CPT1A)

**Figure 1- source data 1.6:** Original file for the western blot in Figure 1C(anti-β-actin)

**Figure 1- source data 1.7:** Original file for the western blot in Figure 1C(anti-CPT1A)

**Figure 1- source data 1.8:** Original file for the western blot in Figure 1C(anti-β-actin)
