# Supplementary material for: CPT1A mediates radiation sensitivity in colorectal cancer
Source: eLife. 2024 Nov 28;13:RP97827. doi: 10.7554/eLife.97827 (PMC11604221; doi:10.7554/eLife.97827)
Supplement: Figure 6—source data 2. [file elife-97827-fig6-data2.zip › Figure 6- source data legends.docx]

**Figure 6- source data 1:** Western blots labelled with relevant bands analyzed in Figure 6A (anti-FOXM1, anti-CPT1A, anti-CAT, anti-SOD3, anti-SOD2, anti-SOD1 and anti-β-actin)

**Figure 6- source data 1.1:** Original file for the western blot in Figure 6A (anti-FOXM1)

**Figure 6- source data 1.2:** Original file for the western blot in Figure 6A (anti-FOXM1)

**Figure 6- source data 1.3:** Original file for the western blot in Figure 6A (anti-CPT1A)

**Figure 6- source data 1.4:** Original file for the western blot in Figure 6A (anti-CPT1A)

**Figure 6- source data 1.5:** Original file for the western blot in Figure 6A (anti-CAT)

**Figure 6- source data 1.6:** Original file for the western blot in Figure 6A (anti-CAT)

**Figure 6- source data 1.7:** Original file for the western blot in Figure 6A (anti-SOD3)

**Figure 6- source data 1.8:** Original file for the western blot in Figure 6A (anti-SOD3)

**Figure 6- source data 1.9:** Original file for the western blot in Figure 6A (anti-SOD2)

**Figure 6- source data 1.10:** Original file for the western blot in Figure 6A (anti-SOD1)

**Figure 6- source data 1.11:** Original file for the western blot in Figure 6A (anti-SOD1)

**Figure 6- source data 1.12:** Original file for the western blot in Figure 6A (anti-β-actin)

**Figure 6- source data 2:** Western blots labelled with relevant bands analyzed in Figure 6D (anti-FOXM1, anti-CPT1A, anti-CAT, anti-SOD2, anti-SOD1 and anti-β-actin)

**Figure 6- source data 2.1:** Original file for the western blot in Figure 6D (anti- FOXM1)

**Figure 6- source data 2.2:** Original file for the western blot in Figure 6D (anti- CPT1A)

**Figure 6- source data 2.3:** Original file for the western blot in Figure 6D (anti- CAT)

**Figure 6- source data 2.4:** Original file for the western blot in Figure 6D (anti- SOD2)

**Figure 6- source data 2.5:** Original file for the western blot in Figure 6D (anti- SOD1)

**Figure 6- source data 2.6:** Original file for the western blot in Figure 6D (anti-β-actin)
